# Arvanil reverses cisplatin resistance in ovarian cancer by activating HMOX1-driven ferroptosis

**DOI:** 10.1038/s41598-026-51046-4

**Published:** 2026-05-02

**Authors:** Zhongping Zhou, Xinglong Liu, Lin Zhao, Nuojie Luo, Yi Sun, Xiangying Deng

**Affiliations:** 1 Institute of Medical Sciences, National Clinical Research Center for Geriatric Diseases (Xiangya Hospital), Central South University, Changsha, 410008 Hunan China; 2 Department of Pathology, The Second Xiangya Hospital, Central South University, Changsha, 410011 Hunan China; 3 Hunan Clinical Medical Research Center for Cancer Pathogenic Genes Testing and Diagnosis, Changsha, 410011 Hunan China; 4 Department of Pathology, Li Xian TCM Hospital, Changde, 415500 Hunan China

**Keywords:** Arvanil, Cisplatin, Cisplatin resistance, Ovarian cancer, Ferroptosis, Cancer, Drug discovery, Oncology

## Abstract

Cisplatin resistance limits the effectiveness of ovarian cancer (OC) therapy. Arvanil is a synthetic capsaicin derivative with favorable pharmacokinetic properties. This study showed that Arvanil enhances the anti-tumor effect of cisplatin in cisplatin-resistant ovarian cancer cells, partly associated with HMOX1-related ferroptosis. The combination treatment elevated reactive oxygen species (ROS), lipid peroxidation (LPO), and ferrous ion (Fe²⁺) levels, suppressed GPX4 protein expression, and activated multiple key ferroptosis regulators. Inhibition of HMOX1 partially reversed these effects, confirming its pivotal regulatory role. In vivo experiments further validated the synergistic anti-tumor efficacy of the Arvanil-cisplatin combination, with no significant changes in body weight and no apparent histopathological or serum biochemical abnormalities detected under the tested conditions. This study provides a novel strategy to overcome cisplatin resistance and expands the potential application of small-molecule ferroptosis inducers in cancer therapy.

**Supplementary Information:**

The online version contains supplementary material available at 10.1038/s41598-026-51046-4.

## Introduction

Ovarian cancer is one of the most lethal gynecologic malignancies worldwide, with high recurrence rates and the development of chemotherapy resistance being the principal causes of its persistently poor long-term survival outcomes^1,2^. Cisplatin remains a cornerstone chemotherapeutic agent and is initially effective in newly diagnosed patients; however, most patients eventually develop acquired resistance after repeated treatment cycles, leading to tumor progression and therapeutic failure^3–5^. Mechanistically, cisplatin resistance is a highly complex and multifactorial process that encompasses dysregulated DNA damage response and homologous recombination repair, aberrant glutathione metabolism, enhanced drug efflux with reduced intracellular accumulation, adaptive remodeling of ROS-antioxidant homeostasis, evasion of cell death programs, and influences from the tumor microenvironment^6–10^. The coordinated interplay of these mechanisms enables tumor cells to withstand sustained chemotherapeutic stress, highlighting the urgent need for chemosensitization strategies that concurrently target multiple resistance pathways rather than single molecular nodes.

In recent years, ferroptosis, an atypical form of programmed cell death, has attracted increasing attention in cancer therapy research^11,12^. Distinct from apoptosis or autophagy, ferroptosis is characterized by iron-dependent LPO and the accumulation of intracellular free iron, ultimately resulting in irreversible disruption of cell membrane integrity. Accumulating evidence indicates that cisplatin-resistant tumor cells often acquire enhanced ferroptosis-evading capacity, for example through upregulation of GPX4 or SLC7A11 to detoxify lipid peroxides and thereby escape drug-induced cell death^13,14^. Accordingly, therapeutic strategies aimed at inducing ferroptosis have emerged as a promising approach to overcome cisplatin resistance.

Capsaicin, the major bioactive constituent of Capsicum species, exhibits antioxidant, antiproliferative, and antitumor activities^15^. In multiple cancer models, capsaicin has been reported to sensitize tumor cells to chemotherapeutic agents by promoting ROS generation and mitochondrial dysfunction, thereby engaging ferroptosis-related pathways^16^. However, the clinical application of capsaicin is substantially limited by its poor bioavailability and dose-limiting side effects. Arvanil is a synthetic capsaicin analog that incorporates the vanillyl amide moiety of capsaicin and displays partial affinity for the cannabinoid CB1 receptor, resulting in improved chemical stability and pharmacological properties. Previous studies have shown that Arvanil exerts pronounced antiproliferative and pro-death effects in models of neurodegenerative diseases and selected solid tumors^17^. Nevertheless, whether Arvanil can overcome cisplatin resistance in OC through activation of ferroptosis pathways has not yet been systematically investigated.

In this study, we explored the potential of Arvanil to reverse cisplatin resistance in OC by inducing ferroptosis and elucidated the underlying molecular mechanisms. Using cisplatin-resistant OC cell lines and in vivo xenograft models, we evaluated the antitumor efficacy of Arvanil alone or in combination with cisplatin and examined its regulatory effects on key ferroptosis-associated factors. Our findings identify a small-molecule, natural-derivative-based ferroptosis-activating strategy that may broaden the therapeutic landscape of chemosensitization and facilitate the translational development of Arvanil in precision oncology.

## Results

### Arvanil potentiates cisplatin cytotoxicity in cisplatin-resistant OC cells

The average IC₅₀ values for A2780 and SKOV3 cells were 4.35 µM and 11.42 µM, respectively, while those for the cisplatin-resistant A2780-DDP and SKOV3-DDP cells were significantly higher, at 24.36 µM and 51.06 µM, respectively (Table 1). The calculated resistance indices (RI) for A2780-DDP and SKOV3-DDP cells were 5.60 and 4.47, respectively.

**Table 1:** The IC50 values of cisplatin/Arvanil in the parental and cisplatin-resistant OC cell lines.

Cell lines	IC50 of cisplatin	RI	IC50 of Arvanil	RI
A2780	4.35 ± 0.32	5.60	1.12 ± 0.17	1.11
A2780-DDP	24.36 ± 2.04		1.25 ± 0.19	
SKOV3	11.42 ± 0.28	4.47	1.64 ± 0.03	1.18
SKOV3-DDP	51.06 ± 2.13		1.94 ± 0.12	

RI, resistance index. IC₅₀ values (µM) are presented as mean ± SD from three independent experiments (*n* = 3) and were determined by nonlinear regression analysis.

Notably, the IC₅₀ values of Arvanil in the resistant cell lines were comparable to those observed in their parental counterparts (Table 1 and Fig. S1), indicating that these cisplatin-resistant OC cells do not exhibit cross-resistance to Arvanil.

**Fig. 1:**
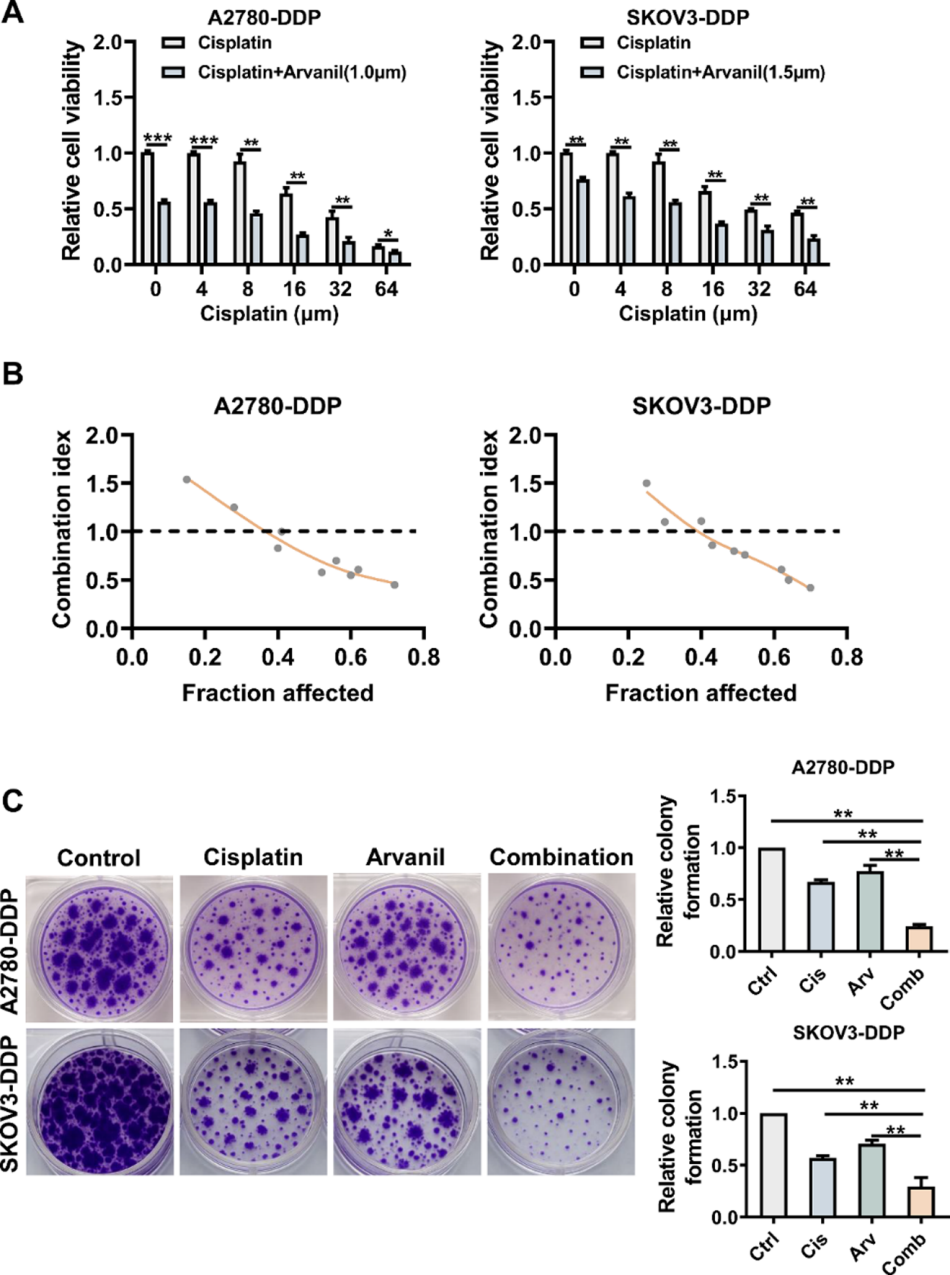
Arvanil enhances the cytotoxic effect of cisplatin in cisplatin-resistant OC cells. **(A)** Cell viability was measured after 48 h of treatment with the indicated concentrations of cisplatin, Arvanil, or their combination. **(B)** Drug synergy was assessed by calculating combination index (CI) values using CompuSyn software. The “fraction affected” (Fa) represents the proportion of inhibited cells, while CI values indicate the type of interaction: CI < 1 indicates synergy, CI = 1 denotes an additive effect, and CI > 1 suggests antagonism. **(C)** Colony formation assays were performed after 14 days of drug treatment to evaluate long-term proliferative capacity. *p < 0.05; **p < 0.01; ***p < 0.001; ****p < 0.0001; ns, *p* > 0.05..

Preliminary experiments suggested that Arvanil could sensitize cisplatin-resistant OC cells to cisplatin (Fig. 1A and Fig. S2). Notably, because Arvanil at 1.0 µM displayed moderate cytotoxicity as a single agent, we further validated the interaction using a broader dose matrix, including lower Arvanil concentrations that are close to the IC_25_ range (Fig. S3-S4). Cell viability assays performed after 48 h of treatment showed that co-treatment with Arvanil consistently potentiated the cytotoxic effects of cisplatin across multiple concentration combinations. To quantitatively evaluate drug interactions independent of single-agent effects, combination index (CI) values were calculated using the Chou-Talalay method (CompuSyn). CI analysis indicated synergistic interactions (CI < 1) at selected concentration combinations (Fig. 1B and Supplementary 1). In A2780-DDP cells, 7.0 µM cisplatin combined with 1.0 µM Arvanil yielded a CI value of 0.45, while in SKOV3-DDP cells, 12.0 µM cisplatin combined with 1.5 µM Arvanil yielded a CI value of 0.42. Consistently, the combination at these concentrations significantly reduced colony formation compared with either agent alone (Fig. 1C). Collectively, these results indicate that Arvanil enhances cisplatin cytotoxicity in cisplatin-resistant OC cells, with CI analysis supporting synergistic interactions at specific dose combinations rather than effects driven by Arvanil alone.

### RNA sequencing analysis revealed that the combination treatment induced the activation of ferroptosis-related pathways

**Fig. 2:**
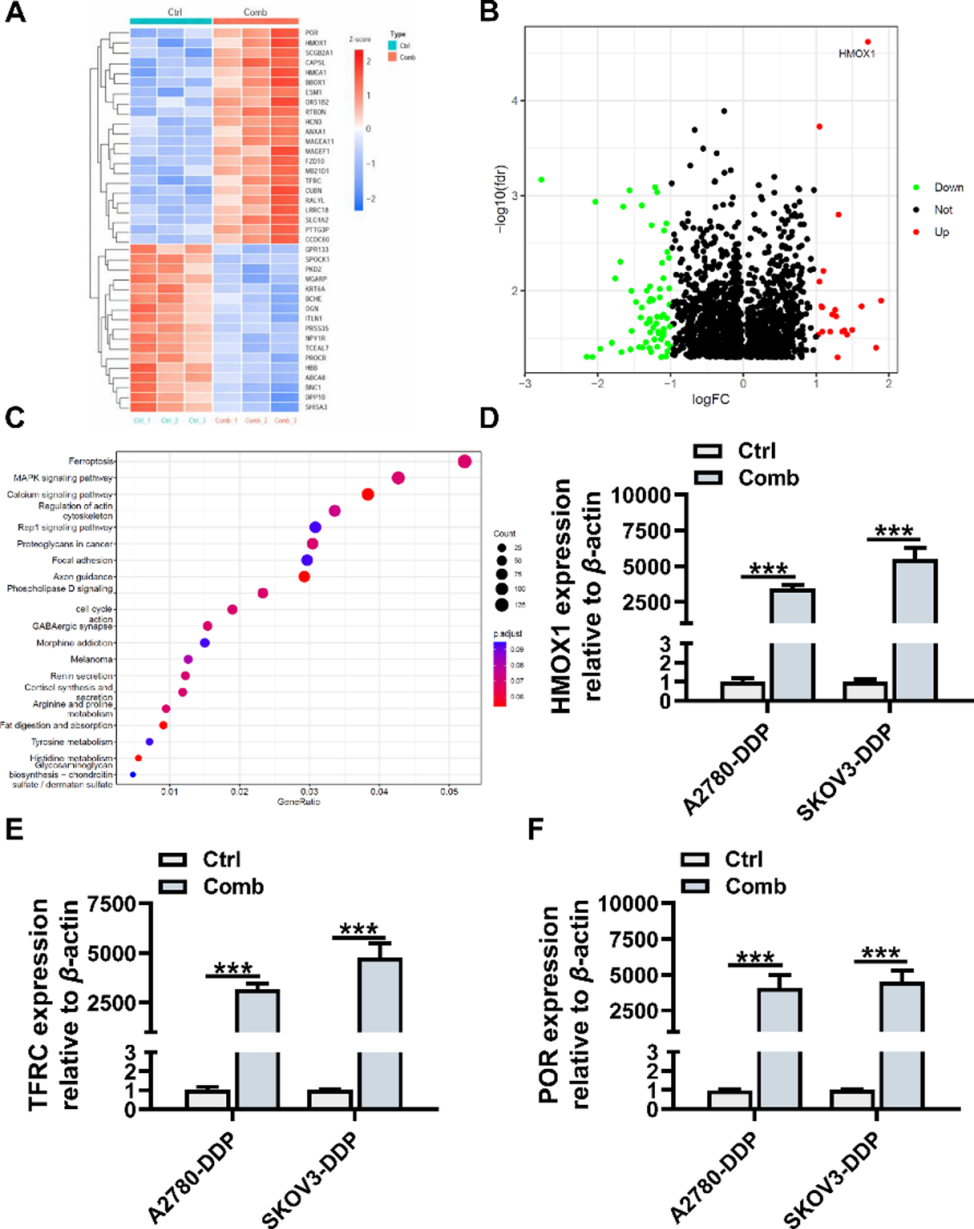
Transcriptomic analysis reveals ferroptosis-related gene activation following combination treatment. Heatmap showing differentially expressed genes (DEGs) between control and combination treatment groups in A2780-DDP cells, based on hierarchical clustering of Z-score normalized expression. **(B)** Volcano plot displaying significantly upregulated and downregulated genes in the combination treatment group. **(C)** Pathway enrichment analysis of differentially expressed genes performed using bioinformatics tools, highlighting activation of ferroptosis-related pathways. **(D-F)** qRT-PCR validation of HMOX1, TFRC, and POR expression in A2780-DDP and SKOV3-DDP cells after 48 h of treatment with the indicated drug concentrations. *p < 0.05; **p < 0.01; ***p < 0.001; ****p < 0.0001; ns, *p* > 0.05..

To elucidate the molecular mechanisms underlying the enhanced cytotoxicity of the combination therapy in cisplatin-resistant OC cells, RNA sequencing was performed on A2780-DDP cells, and differential expression analysis was primarily conducted by comparing the combination treatment group with the untreated control. Heatmap and volcano plot analyses identified significantly upregulated and downregulated genes in response to the combination treatment (Fig. 2A-B), among which HMOX1 showed notably increased expression.

Subsequent bioinformatics analysis of the differentially expressed genes revealed significant enrichment and activation of ferroptosis-related pathways in the combination group (Fig. 2C). These transcriptomic findings were further validated by quantitative real-time PCR (qRT-PCR), which confirmed the upregulation of key ferroptosis-associated genes, including HMOX1, TFRC, and POR (Fig. 2D-F).

Collectively, these results indicate that Arvanil enhances the anti-tumor efficacy of cisplatin in resistant OC cells at least in part by promoting ferroptosis.

### Arvanil promotes cisplatin-induced ferroptosis in cisplatin-resistant OC cells

**Fig. 3:**
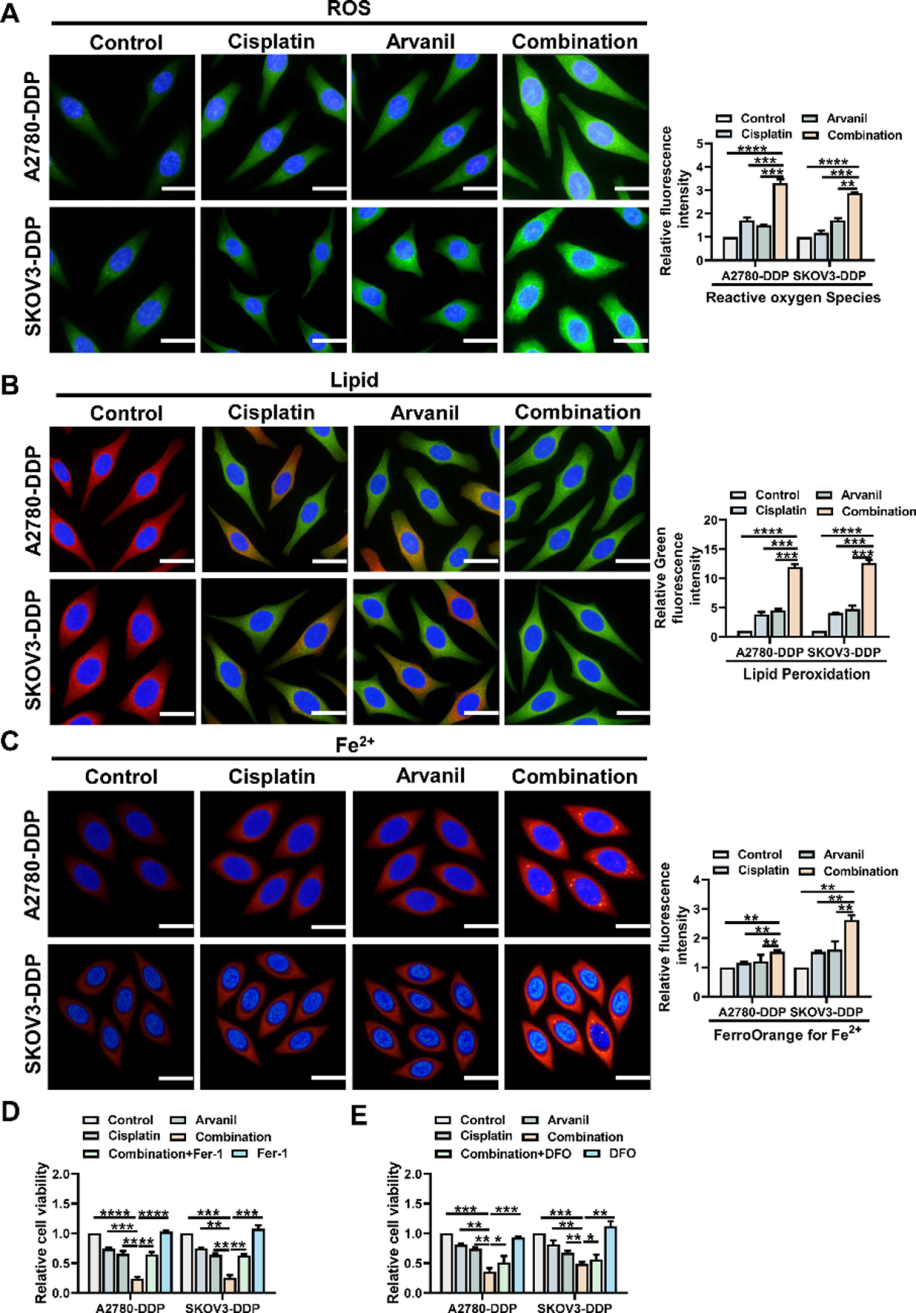
Combination treatment induces ferroptosis in cisplatin-resistant OC cells. **(A)** Intracellular ROS levels following drug treatment. **(B)** Intracellular LPO levels in response to the indicated treatments. **(C)** Intracellular ferrous iron (Fe²⁺) levels measured after drug exposure. **(D-E)** Effect of ferroptosis inhibitors, deferoxamine (DFO) and ferrostatin-1 (Fer-1), on cell viability in the combination treatment group. *p < 0.05; **p < 0.01; ***p < 0.001; ****p < 0.0001; ns, *p* > 0.05.

Ferroptosis is a distinct form of regulated cell death characterized by the accumulation of ROS and LPO. Based on the RNA-seq results, we further evaluated ROS and LPO levels in cisplatin-resistant OC cells. The combination treatment significantly increased both intracellular ROS and LPO levels compared to single-agent treatments (Fig. 3A-B). In addition, FerroOrange staining revealed a marked elevation in intracellular Fe²⁺ levels—a hallmark of ferroptosis—in the combination group (Fig. 3C).

To verify the involvement of ferroptosis, we employed the ferroptosis inhibitor ferrostatin-1 (Fer-1) and the iron chelator deferoxamine (DFO). Both agents effectively reversed the cytotoxic effects of the combination treatment on cell viability (Fig. 3D-E), supporting the conclusion that ferroptosis plays a critical role in mediating the observed anti-tumor response.

**Fig. 4:**
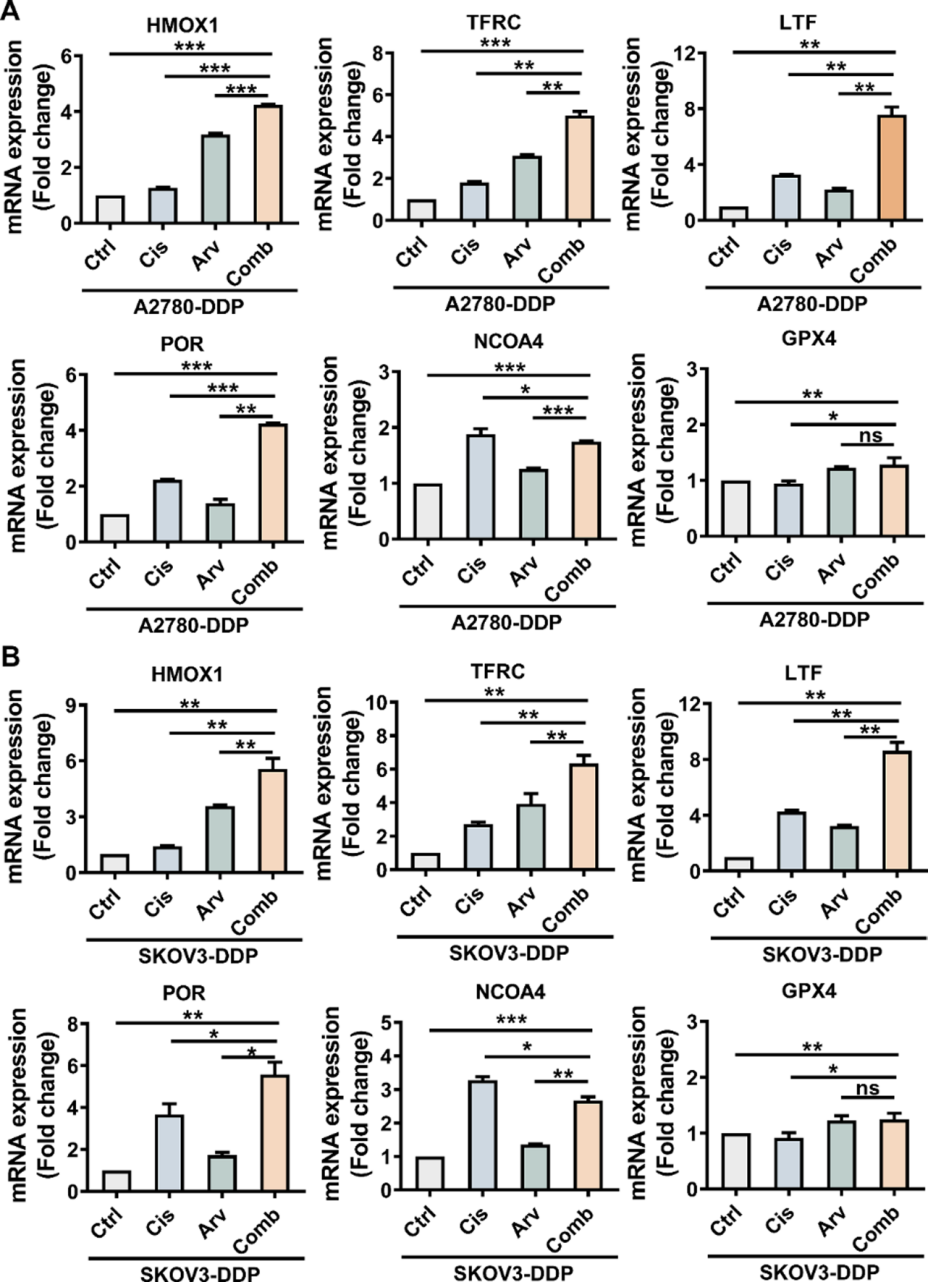
Combination treatment upregulates ferroptosis-associated genes in A2780-DDP and SKOV3-DDP cells. **(A-B)** Relative mRNA expression levels of key ferroptosis-related genes following treatment with cisplatin, Arvanil, or their combination. *p < 0.05; **p < 0.01; ***p < 0.001; ****p < 0.0001; ns, *p* > 0.05..

**Fig. 5:**
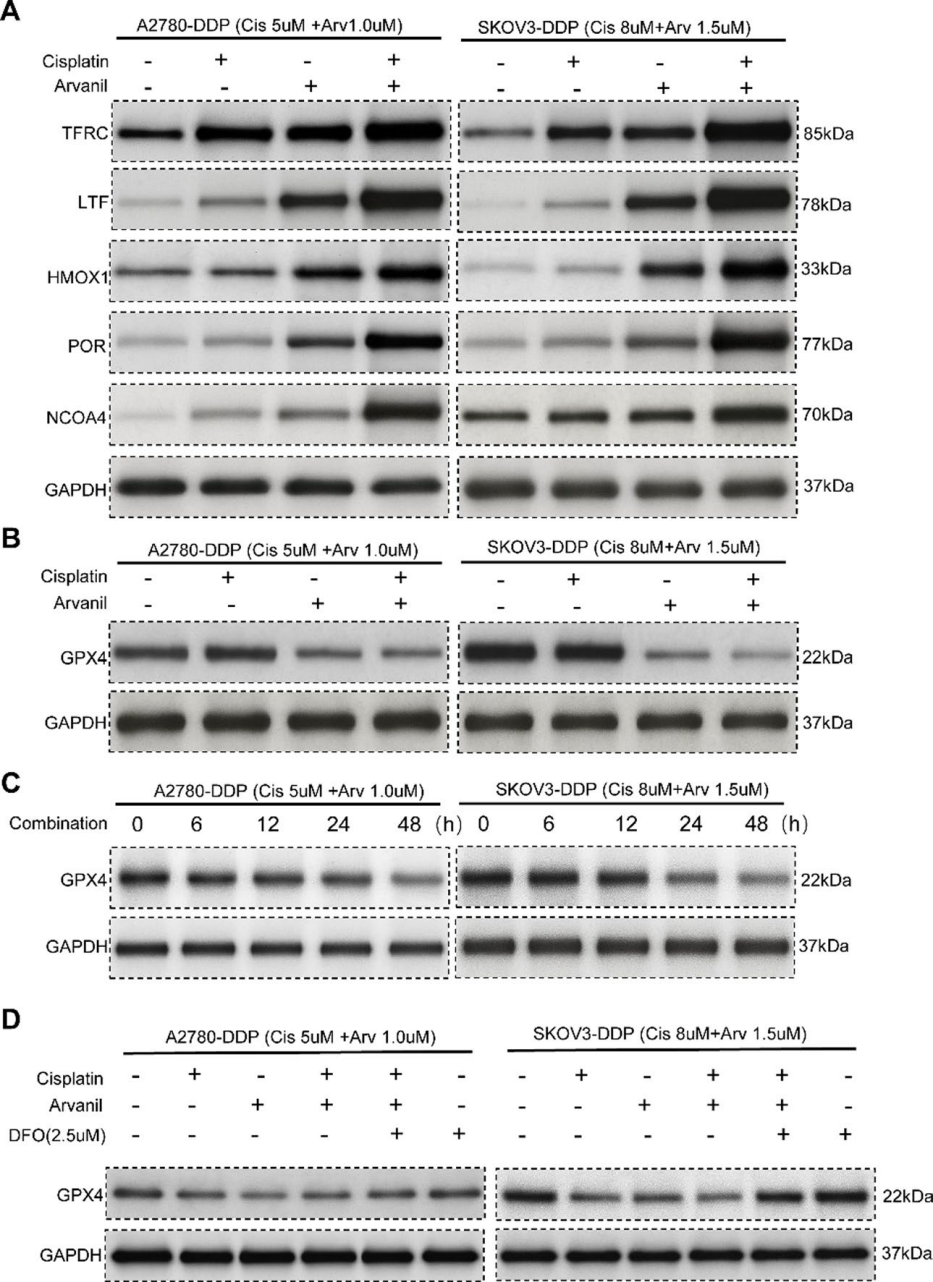
Combination treatment induces ferroptosis-related protein expression in A2780-DDP and SKOV3-DDP cells. **(A)** Western blot analysis of ferroptosis-associated proteins following drug treatment. **(B)** GPX4 protein levels after 48 h of treatment with cisplatin, Arvanil, or their combination. **(C)** Time-dependent changes in GPX4 expression following combination treatment. **(D)** Effect of the ferroptosis inhibitor deferoxamine (DFO) on GPX4 protein levels after 48-hour treatment. All uncropped blots are provided in Supplementary 2.

Next, we analyzed the expression of key ferroptosis-related genes, including TFRC, LTF, HMOX1, POR, NCOA4, and GPX4, at both the mRNA and protein levels following drug treatment. The combination therapy markedly upregulated the expression of TFRC, LTF, HMOX1, POR, and NCOA4 (Figs. 4A-B and 5A and Fig. S5A). Interestingly, although GPX4 mRNA expression remained largely unchanged, GPX4 protein levels were substantially reduced (Fig. 5B and Fig. S5B), which may involve post-transcriptional regulation or feedback mechanisms.

A time-course analysis revealed a gradual decrease in GPX4 protein levels following combination treatment (Fig. 5C and Fig. S5C), which was reversed by DFO administration (Fig. 5D and Fig. S5D), indicating that Fe²⁺ accumulation mediates GPX4 suppression.

Taken together, these findings provide compelling evidence that the combination of Arvanil and cisplatin induces ferroptosis in cisplatin-resistant OC cells through iron accumulation and modulation of ferroptosis-related gene expression.

### Combination treatment is associated with HMOX1-related ferroptosis and impaired DNA damage repair in cisplatin-resistant OC cells

**Fig. 6:**
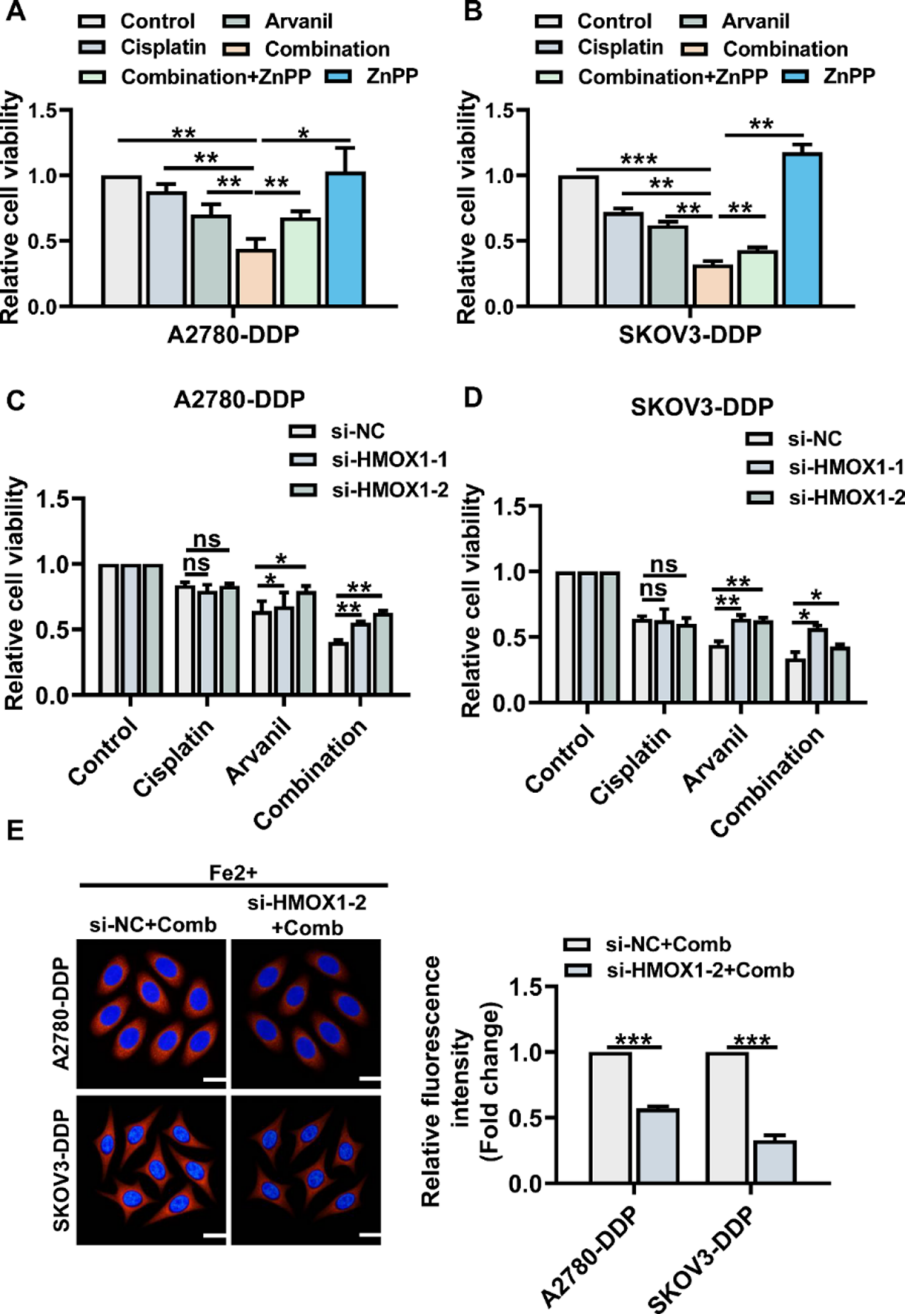
Combination treatment induces ferroptosis via HMOX1 upregulation. **(A–B)** Effect of HMOX1 inhibition by zinc protoporphyrin IX (ZnPP, 50 nM) on cell viability after 48 h of drug treatment. **(C–D)** Effect of HMOX1 knockdown using siRNAs (20 nM) on cell viability following 48-hour treatment. **(E)** Impact of HMOX1 silencing on intracellular Fe²⁺ levels in cells treated with cisplatin and Arvanil. *p < 0.05; **p < 0.01; ***p < 0.001; ****p < 0.0001; ns, *p* > 0.05..

Overexpression of HMOX1 has been reported to promote ferroptosis by enhancing pro-oxidant activity^18^. To investigate the role of HMOX1 in ferroptosis induced by the combination treatment, we employed both pharmacological inhibition and siRNA-mediated gene silencing approaches. Inhibition of HMOX1 with zinc protoporphyrin IX (ZnPP), as well as HMOX1 knockdown via siRNA, significantly restored cell viability and reduced cell death triggered by the combination of Arvanil and cisplatin (Fig. 6A-D). Furthermore, silencing of HMOX1 markedly decreased intracellular Fe²⁺ levels, as measured by FerroOrange staining (Fig. 6E).

These findings indicate that HMOX1 contributes to Fe²⁺ accumulation and ferroptosis in cisplatin-resistant OC cells in response to combination therapy, and support a role for HMOX1 upregulation in the ferroptosis response induced by Arvanil and cisplatin co-treatment.

Given that enhanced DNA damage repair capacity is an important mechanism underlying the development of cisplatin resistance, we further evaluated the effects of Arvanil on DNA damage accumulation and homologous recombination (HR) repair. Cisplatin-resistant OC cells were treated with cisplatin, Arvanil, or their combination, followed by immunofluorescence staining of γH2AX and RAD51 to assess DNA damage and HR repair status. The results showed that Arvanil treatment markedly increased γH2AX foci formation while suppressing RAD51 foci formation, indicating enhanced DNA damage accumulation and impaired HR repair capacity. Notably, these effects were further amplified upon co-treatment with cisplatin (Fig. S6).

### Combination treatment inhibits xenograft tumor growth in vivo

To assess the potential cytotoxicity of the combination regimen toward normal cells, preliminary in vitro experiments were first conducted using human normal ovarian epithelial IOSE80 cells. Cell viability was evaluated by CCK-8 assay following treatment with cisplatin, Arvanil, or their combination. The results showed that the combination treatment did not induce significant cytotoxic effects in IOSE80 cells (Fig.S7), indicating that Arvanil enhances the anti-tumor activity of cisplatin without increasing toxicity to normal ovarian epithelial cells.

**Fig. 7:**
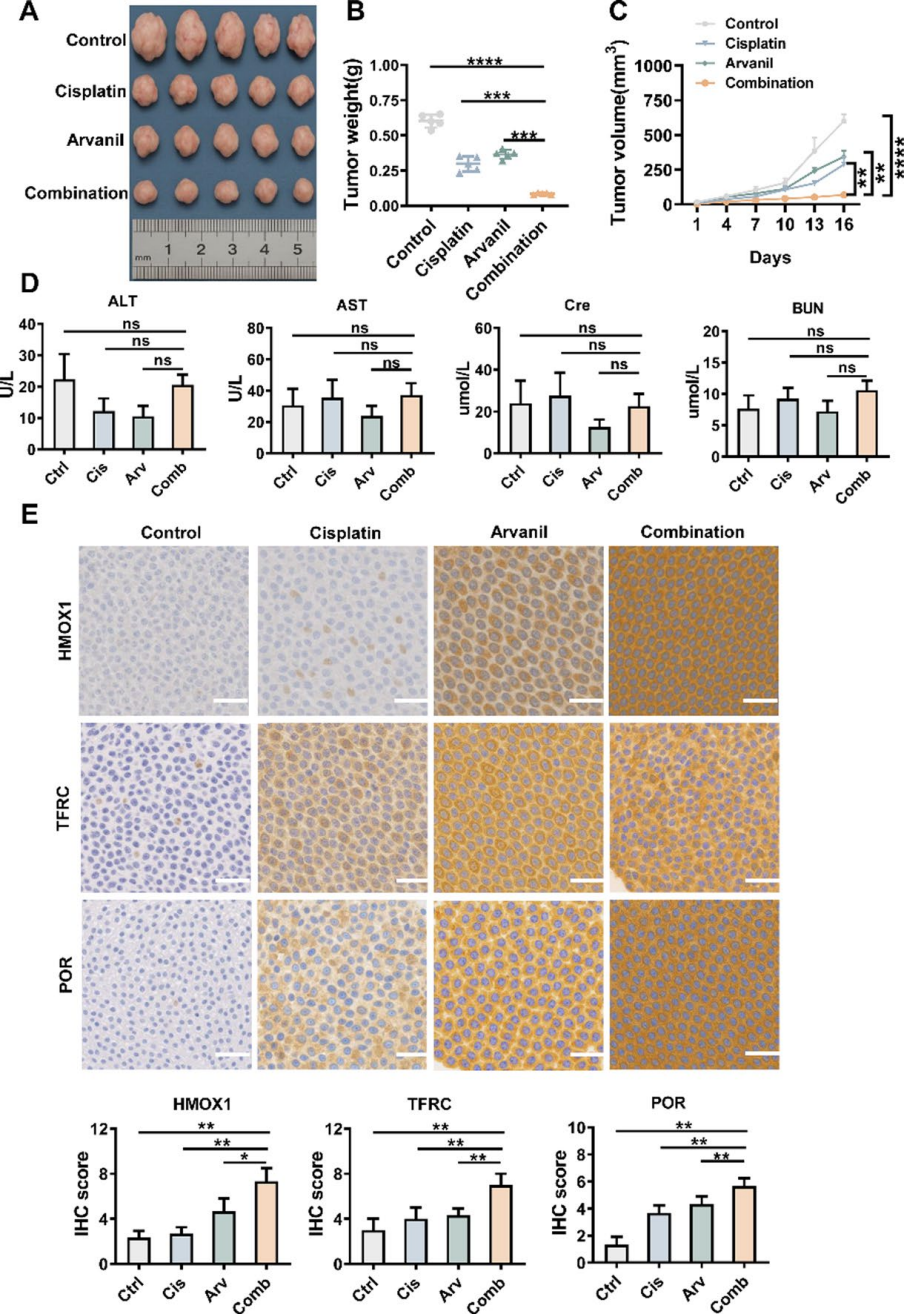
Combination treatment suppresses xenograft tumor growth in vivo. **(A)** Representative images of xenograft tumors harvested from mice after 16 days of treatment with cisplatin, Arvanil, or their combination. **(B)** Tumor weight measured at the end of the treatment period. **(C)** Tumor volume progression during treatment. **(D)** Serum levels of alanine aminotransferase (ALT), aspartate aminotransferase (AST), blood urea nitrogen (BUN), and creatinine (CRE) after 16 days of treatment, indicating hepatic and renal function. **(E)** Immunohistochemical analysis of xenograft tumor tissues, showing expression of ferroptosis-related proteins. *p < 0.05; **p < 0.01; ***p < 0.001; ****p < 0.0001; ns, *p* > 0.05.

Subsequently, the in vivo anti-tumor efficacy of the combination treatment was evaluated using a xenograft model established with A2780-DDP cells. Mice bearing subcutaneous tumors were treated with intraperitoneal injections of cisplatin (3.0 mg/kg) and Arvanil (10 mg/kg), administered either individually or in combination every three days, with saline-treated mice serving as the control group. Compared with either monotherapy or the control group, the combination treatment significantly inhibited tumor growth and reduced tumor volume (Fig. 7A-C).

Importantly, the combination regimen did not show evidence of systemic toxicity in vivo under the tested conditions. No significant differences in body weight were observed among the treatment groups throughout the experimental period, indicating good systemic tolerability (Fig. S8A). Consistently, histopathological examination of major organs, including the heart, liver, spleen, lung, and kidney, revealed no evident tissue damage or morphological abnormalities (Fig. S8B). In addition, serum biochemical analysis showed no significant differences in ALT, AST, CRE, or BUN levels among the groups, further supporting the absence of apparent hepatic or renal toxicity (Fig. 7D).

Mechanistically, immunohistochemical staining demonstrated elevated expression of ferroptosis-related proteins, including HMOX1, TFRC, and POR, in tumor tissues from the combination treatment group (Fig. 7E). Notably, a marked increase in 4-hydroxynonenal (4-HNE), a lipid peroxidation marker, was also observed following combination treatment (Fig. S8C-D), indicating enhanced lipid peroxidation in vivo.

Collectively, these findings support a potential synergistic anti-tumor effect of Arvanil and cisplatin in vivo in cisplatin-resistant OC, partially associated with ferroptosis induction, without evidence of significant systemic toxicity under the tested conditions.

**Fig. 8:**
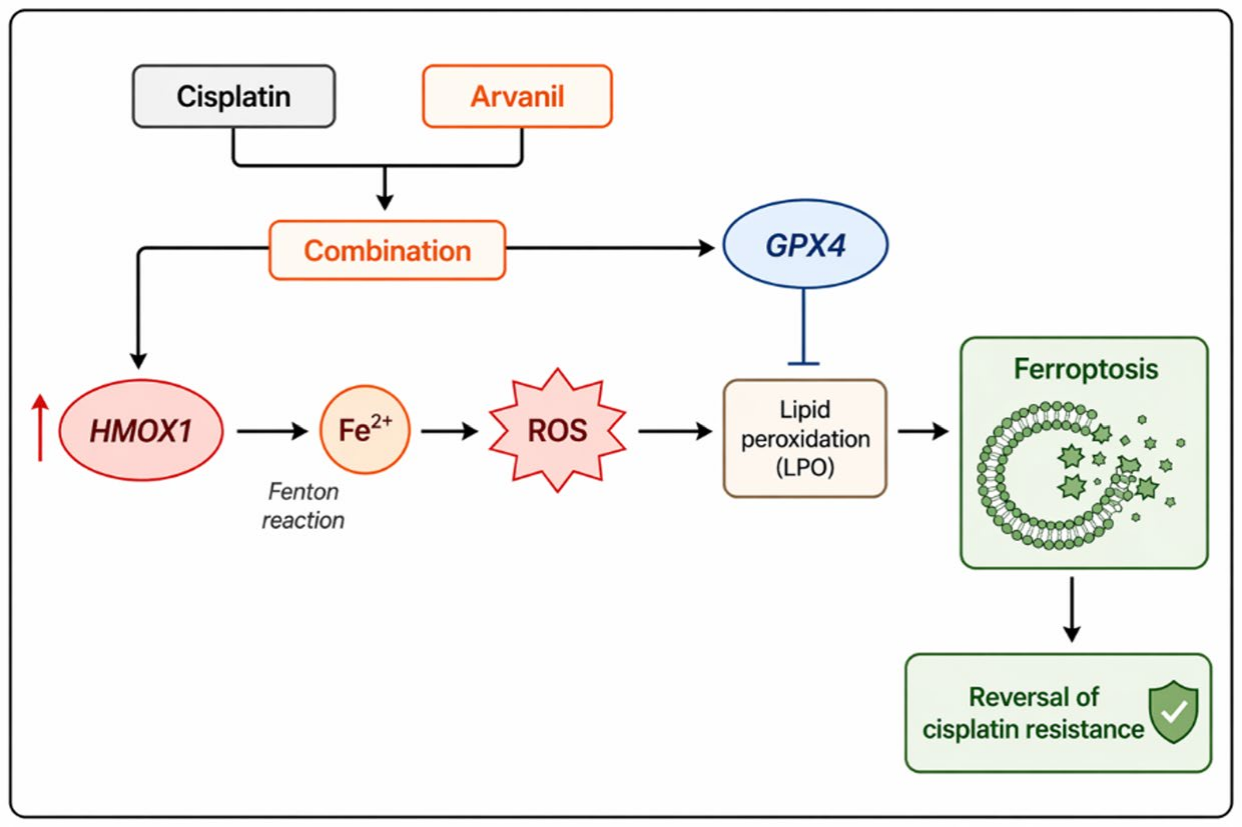
Schematic illustration of the mechanism by which Arvanil sensitizes cisplatin-resistant OC cells to cisplatin. Arvanil promotes HMOX1-dependent ferroptosis by inducing Fe²⁺ accumulation, ROS production, and LPO. These coordinated effects collectively enhance the cytotoxic efficacy of cisplatin in cisplatin-resistant OC cells.

Based on these in vitro and in vivo findings, a schematic model illustrating the proposed mechanism by which Arvanil sensitizes cisplatin-resistant OC cells to cisplatin is presented (Fig. 8).

## Discussion

Chemoresistance remains one of the major causes of treatment failure in advanced ovarian cancer^19^. Increasing evidence indicates that cisplatin resistance is not confined to classical mechanisms such as enhanced drug efflux, increased DNA repair capacity, or altered glutathione metabolism, but rather reflects a systemic adaptive state characterized by profound reprogramming of cellular stress responses and susceptibility to cell death. In particular, cisplatin-resistant cells often acquire broad tolerance to non-apoptotic cell death modalities and intrinsically evade ferroptosis by sustaining GPX4 activity, limiting iron accumulation, and efficiently clearing lipid ROS^20,21^. Accordingly, restoring ferroptotic vulnerability is considered a highly attractive strategy to overcome chemoresistance.

In this study, we demonstrate that the synthetic capsaicin derivative Arvanil markedly enhances cisplatin cytotoxicity in resistant OC cells primarily through reactivation of ferroptosis. Unlike canonical ferroptosis inducers that directly inhibit GPX4 (such as RSL3 or FIN56), Arvanil disrupts ferroptotic defense via an indirect yet amplified mechanism, involving upregulation of HMOX1, promotion of heme degradation–derived iron release, enhancement of LPO, and concomitant reduction of GPX4 protein levels. Through coordinated regulation of iron metabolism and oxidative stress, this process constitutes a multi-layered amplification of ferroptosis, which may provide a more sustained and potentially better-tolerated chemosensitization strategy in resistant tumors.

One possible mechanism underlying the observed effects may involve the context-dependent functional switch of HMOX1. As a classical stress-inducible enzyme, HMOX1 exerts cytoprotective effects under moderate activation by degrading pro-oxidant heme and alleviating oxidative stress; however, under sustained or excessive activation, it promotes heme degradation and releases large amounts of free iron, leading to iron overload and triggering iron-dependent cell death^22,23^. Notably, there is no fixed " expression threshold " for HMOX1; rather, its functional transition is governed by a " functional threshold " determined by the intensity of intracellular oxidative stress and the burden of iron metabolism^24^. In the present study, the combination of Arvanil and cisplatin markedly increased ROS levels and disrupted redox homeostasis. Under this high oxidative stress condition, HMOX1 was upregulated and accelerated heme degradation, resulting in Fe²⁺ accumulation. Excess Fe²⁺ further amplified lipid peroxidation via the Fenton reaction, while the downregulation of GPX4 impaired the cellular capacity to detoxify lipid peroxides. Collectively, these alterations may drive cells beyond their antioxidant buffering capacity, potentially shifting HMOX1 from a protective factor toward a pro-ferroptotic role and contributing to a loop of " iron release-lipid peroxidation-antioxidant imbalance. “.

This context-dependent functional shift may help explain the apparent paradox of the NRF2/HMOX1 pathway in cisplatin resistance. Under low to moderate stress conditions, this pathway promotes cell survival by enhancing antioxidant capacity^23^; however, under the high ROS and iron-overload conditions established in this study, its sustained activation instead leads to uncontrolled iron release and excessive lipid peroxidation, shifting from an adaptive protective response to a pro-death signal. Furthermore, although siRNA-mediated knockdown and pharmacological inhibition using ZnPP confirmed the necessity of HMOX1 in combination treatment-induced ferroptosis, whether HMOX1 alone is sufficient to trigger ferroptosis remains unclear. Current evidence suggests that HMOX1 more likely functions as an amplifier of ferroptosis, with its pro-death effects depending on the synergistic interaction between oxidative stress and impaired antioxidant defenses. Therefore, future studies are required to further define its sufficiency and dynamic threshold characteristics in ferroptosis through HMOX1 overexpression models combined with modulation of oxidative stress levels.

Beyond ferroptosis, Arvanil-induced oxidative stress may also contribute to cisplatin sensitization by exacerbating ROS-associated DNA damage and interfering with homologous recombination (HR) repair. This hypothesis is supported by the observed changes in γH2AX and RAD51 foci; however, further mechanistic studies are required to clarify the relative contribution of DNA damage–repair disruption to overall chemosensitization.

Notably, although GPX4 mRNA levels remain unchanged, GPX4 protein expression is markedly reduced following combination treatment, suggesting the involvement of post-transcriptional regulation or protein degradation pathways, such as the proteasome or autophagy–lysosome system. As the present study primarily focuses on the functional association between ferroptosis induction and chemosensitization, the precise molecular mechanisms underlying GPX4 protein destabilization warrant further investigation.

Although Arvanil is recognized as an agonist of the transient receptor potential vanilloid 1 (TRPV1)^25,26^, the upstream mechanisms underlying its induction of ferroptosis in this study remain to be fully elucidated. Previous studies have shown that Arvanil exerts multi-target effects and that its biological activities are not entirely dependent on TRPV1-mediated signaling^26,27^. In the present study, the combination of Arvanil and cisplatin consistently induced HMOX1 upregulation, Fe²⁺ accumulation, and enhanced lipid peroxidation, all of which were reversed by ferroptosis inhibitors. These findings suggest that its pro-ferroptotic effect is primarily mediated through the “oxidative stress–iron metabolism–lipid peroxidation " axis, which may function independently of specific membrane receptor signaling.

On the other hand, as a Ca²⁺-permeable channel, TRPV1 may be involved in this process through regulation of intracellular Ca²⁺ homeostasis and ROS generation; however, its precise contribution has not been directly examined in this study. Therefore, whether TRPV1 is involved in Arvanil-induced ferroptosis warrants further investigation. Future studies incorporating specific TRPV1 antagonists (e.g., Capsazepine) or genetic intervention approaches will be necessary to clarify its role.

From a translational perspective, small-molecule agents such as Arvanil offer advantages including structural simplicity, chemical stability, and favorable druggability. However, ferroptosis induction is intrinsically context-dependent, and its therapeutic window may be jointly determined by iron availability, lipid oxidative burden, and antioxidant buffering capacity^28,29^. Moreover, ferroptosis-associated membrane rupture can lead to the release of damage-associated molecular patterns (DAMPs), thereby modulating tumor immune responses in a context-dependent manner^30,31^. Although this raises the possibility that ferroptosis induction may synergize with immune checkpoint blockade, such effects have not yet been validated in immunocompetent models.

Encouragingly, in vivo experiments demonstrate that Arvanil combined with cisplatin significantly enhances tumor suppression, with no evident hepatic or renal toxicity observed under the tested conditions. Nevertheless, given the pronounced molecular and histological heterogeneity of OC, conclusions derived from A2780-DDP and SKOV3-DDP models may not be universally applicable. The HMOX1-driven ferroptosis sensitization mechanism proposed here may be particularly relevant to OC subtypes characterized by high oxidative stress burden and iron metabolic reprogramming, such as subsets of high-grade serous OC. Future studies using patient-derived organoids and PDX models will be essential to validate efficacy stability, assess inter-individual variability, and determine whether HMOX1, POR, or TFRC can serve as predictive biomarkers of response to combination therapy.

## Materials and methods

### Antibodies and reagents

The following primary antibodies were used in this study: anti-HMOX1 (ab52947, Abcam), anti-TFRC (ab214039, Abcam), anti-LTF (ab109216, Abcam), anti-POR (ab180597, Abcam), anti-GPX4 (ab125066, Abcam), anti-β-actin (A5441, Sigma-Aldrich), anti-γH2A.X (phospho S139) antibody (ab22551, Abcam), anti-4-hydroxynonenal (4-HNE) antibody (HY-P81208, MCE) and anti-Rad51 antibody (ab133534, Abcam).

Chemical reagents included Arvanil (HY-103333), cisplatin (HY-17394), deferoxamine mesylate (DFO, HY-B0988), ferrostatin-1 (Fer-1, HY-100579), and zinc protoporphyrin (ZnPP, HY-101193), all obtained from MedChemExpress (MCE). Lipid peroxidation and oxidative stress were assessed using BODIPY™ 581/591 C11 (D3861, Thermo Fisher Scientific), a ROS detection kit (S0033S, Beyotime Biotechnology), and FerroOrange (F374, Dojindo Laboratories). Cell viability was measured using Cell Counting Kit-8 (CK04, Dojindo). siRNAs were purchased from RiboBio (Guangzhou, China), and transfections were performed using Lipofectamine™ 3000 (L3000015, Invitrogen).

Biochemical assay kits for alanine aminotransferase (ALT, C009-2-1), aspartate aminotransferase (AST, C010-2-1), blood urea nitrogen (BUN, C013-2-1), and creatinine (CRE, C011-2-1) were obtained from Nanjing Jiancheng Bioengineering Institute (Nanjing, China).

## Cell culture

The human ovarian cancer cell lines A2780, SKOV3, OV-90, and OVCAR-8, as well as the human normal ovarian epithelial cell line IOSE80, were obtained from the National Collection of Authenticated Cell Cultures (Shanghai, China). Cisplatin-resistant sublines (A2780-DDP and SKOV3-DDP) were established in our laboratory by stepwise exposure to increasing concentrations of cisplatin. Resistant cells were routinely maintained in medium containing a low dose of cisplatin to preserve the resistant phenotype, and cisplatin was withdrawn for 5–7 days before all functional assays. All cell lines, including A2780, SKOV3, A2780-DDP, SKOV3-DDP, OV-90, OVCAR-8, and IOSE80, were cultured in Dulbecco’s Modified Eagle Medium (DMEM; Sigma, Cat# D5796) supplemented with 10% fetal bovine serum (FBS) and 1% penicillin–streptomycin (P1400, Solarbio). All cells were maintained at 37 °C in a humidified incubator with 5% CO₂.

### CCK-8

OC cells were seeded into 96-well plates and allowed to adhere for 12 h. After treatment with the indicated drugs, the culture medium was replaced with 100 µL of fresh medium containing 10 µL of Cell Counting Kit-8 (CCK-8) reagent. Following a 2-hour incubation at 37 °C, the absorbance at 450 nm was measured using a microplate reader.

### Clone formation assay

Cisplatin-resistant OC cells were seeded into 6-well plates and allowed to adhere. The culture medium was then replaced with fresh medium containing the indicated drug. After a two-week incubation, the medium was removed, and the cells were gently washed twice with phosphate-buffered saline (PBS). Cells were fixed with 4% paraformaldehyde for 15 min and subsequently stained with crystal violet. Following a 30-minute incubation at room temperature (25 °C), excess stain was washed off with double-distilled water (ddH₂O), and the plates were air-dried before analysis.

### RNA sequencing

Total RNA was extracted from A2780-DDP cells treated with the combination of cisplatin and Arvanil, as well as from untreated control cells, using TRIzol reagent (Invitrogen, USA) according to the manufacturer’s protocol. RNA sequencing was performed on the Illumina PE150 platform (HaploX, China). Differentially expressed genes (DEGs) were identified based on a threshold of |log₂ fold change| ≥ 1.

### Detection of ROS

Intracellular ROS levels were assessed using the fluorescent probe DCFH-DA. Cells were seeded in six-well plates and subjected to the indicated treatments. After treatment, cells were gently washed twice with PBS and incubated with 10 µM DCFH-DA in serum-free medium at 37 °C for 30 min in the dark. Cells were then washed three times with PBS to remove excess probe. Fluorescence images were acquired using a laser confocal microscope. For quantitative analysis, all images were captured using identical laser intensity, gain, and exposure settings. At least five randomly selected, non-overlapping fields per sample were analyzed. Mean fluorescence intensity per cell was quantified using Image-Pro Plus 6.0 software by manually delineating regions of interest (ROIs) corresponding to individual cells and subtracting background fluorescence. Data from independent experiments were averaged and normalized to the control group.

### Detection of LPO

The intracellular LPO levels were assessed using the BODIPY fluorescent probe. Treated cells were washed twice with PBS and then incubated with serum-free medium containing 2 µM BODIPY probe at 37 °C for 30 min in the dark. After incubation, cells were washed three times with PBS to remove unbound probe. Fluorescence signals were observed using a laser confocal microscope, where oxidized BODIPY emitted green fluorescence (emission at 510 nm) and reduced BODIPY emitted red fluorescence (emission at 590 nm). The shift in fluorescence intensity reflected the degree of LPO. Oxidized (green) fluorescence was captured using identical imaging parameters for all groups. Quantification was performed by measuring the mean fluorescence intensity per cell using Image-Pro Plus 6.0 software. At least five random fields were analyzed per sample, and fluorescence intensity was normalized to cell number based on DAPI counterstaining. Results were expressed as relative lipid ROS levels compared with the control group.

### Detection of Fe^2+^ levels

Intracellular Fe²⁺ levels were assessed using the FerroOrange fluorescent probe. Following the indicated treatments, cells were washed twice with PBS and incubated with serum-free medium containing 1 µM FerroOrange at 37 °C for 30 min in the dark. Cells were then gently washed three times with PBS to remove excess probe. Fluorescence images were acquired using a laser confocal microscope, and fluorescence intensity was used as an indicator of intracellular Fe²⁺ levels. For quantitative analysis, all images were captured using identical imaging parameters. At least five randomly selected, non-overlapping fields per sample were analyzed. Mean fluorescence intensity per cell was quantified using Image-Pro Plus 6.0 software after background subtraction and normalized to cell number based on DAPI-stained nuclei. Data are presented as the mean ± SD from three independent experiments.

### Detection of γH2AX and RAD51

Cells grown on glass coverslips were treated as indicated, washed with PBS, fixed in 4% paraformaldehyde for 15 min, and permeabilized with 0.2% Triton X-100 for 10 min. After blocking with 5% bovine serum albumin (BSA) for 1 h at room temperature, cells were incubated overnight at 4 °C with primary antibodies against γH2AX (Ser139) and RAD51. Following PBS washes, cells were incubated with Alexa Fluor 488 and Alexa Fluor 594 conjugated secondary antibodies for 1 h in the dark, and nuclei were counterstained with DAPI. Fluorescence images were acquired using a laser confocal microscope under identical acquisition settings. For quantitative analysis, at least five randomly selected, non-overlapping fields were analyzed per group, with 10–15 nuclei evaluated. After background subtraction, nuclear ROIs were defined based on DAPI staining. The fluorescence intensity of γH2AX and RAD51 within nuclear ROIs was quantified using Image-Pro Plus 6.0 software. Mean fluorescence intensity (MFI) per nucleus was calculated and used for statistical analysis. All analyses were performed in a blinded manner.

### qRT-PCR

Total RNA was extracted using TRIzol reagent (Invitrogen, CA, USA) following the manufacturer’s protocol. Subsequently, 1 µg of RNA was reverse transcribed into complementary DNA (cDNA) using the HiScript II Q RT SuperMix for qPCR (Vazyme, Nanjing, China). Quantitative real-time PCR (qRT-PCR) was performed with 2× SYBR Green qPCR Master Mix (Bimake, Changsha, China). All primers were synthesized by Tsingke Biotechnology (Changsha, China), and their sequences are listed in Table S1.

### Western blot

Equal amounts of total protein from each sample were separated by SDS-PAGE and transferred onto polyvinylidene difluoride (PVDF) membranes. The membranes were blocked with non-fat milk to minimize nonspecific binding, followed by incubation with appropriately diluted primary antibodies. After thorough washing, membranes were incubated with horseradish peroxidase (HRP)-conjugated secondary antibodies. Protein signals were detected using an enhanced chemiluminescence (ECL) system in accordance with the manufacturer’s protocol. Uncropped blots are provided in the Supplementary 2.

### siRNA and transfection

Cisplatin-resistant OC cells were seeded into 6-well plates and cultured overnight. Transfection was performed using Lipofectamine™ 3000 (Invitrogen) according to the manufacturer’s instructions. The siRNA sequences used are listed in Table S2.

### Xenograft model

Female BALB/c nude mice (4 weeks old) were purchased from Hunan SJA Laboratory Animal Co., Ltd. (Changsha, China). All animal procedures were approved by the Medical Ethics Committee of Central South University. A total of 6 × 10⁶ A2780-DDP cells suspended in 200 µL of PBS were subcutaneously injected into the right flank of each mouse. Five days post-inoculation, the mice were randomly assigned to four groups (*n* = 5 per group): (1) control, (2) cisplatin, (3) Arvanil, and (4) cisplatin + Arvanil. Cisplatin (3.0 mg/kg) and Arvanil (10 mg/kg) were administered intraperitoneally every three days in a total volume of 100 µL. Body weight and tumor volume were measured every three days. Tumor volume was calculated using the formula: 0.5 × length × width². After 16 days of treatment, the mice were euthanized. Blood samples were collected for biochemical analysis, and tumors were excised, weighed, and fixed in 10% neutral-buffered formalin for further histological evaluation. Individual tumor dimensions (length and width) for each mouse at all measurement time points, final tumor weights, and animal body weights are provided in the Supplementary Information (Supplementary 3). Mice were euthanized by cervical dislocation performed by trained personnel in accordance with institutional animal care guidelines and the ARRIVE guidelines. No chemical agents were used. All animal experiments were approved by the Institutional Animal Care and Use Committee of Central South University and were reported in accordance with the ARRIVE guidelines.

### Biomedical measurement

Serum levels of alanine aminotransferase (ALT), aspartate aminotransferase (AST), blood urea nitrogen (BUN), and creatinine (CRE) were determined using the corresponding assay kits, following the manufacturers’ instructions.

### Hematoxylin and eosin (H&E) staining

Tissue samples were harvested and fixed in 4% paraformaldehyde for 24 h at room temperature. After fixation, the tissues were dehydrated through a graded ethanol series, cleared in xylene, and embedded in paraffin. Paraffin-embedded tissues were sectioned into 4 μm-thick slices using a microtome. The sections were deparaffinized in xylene and rehydrated through a descending ethanol series. Subsequently, the sections were stained with hematoxylin for 5 min, rinsed with running tap water, and differentiated in 1% acid alcohol if necessary. After bluing in running water, the sections were counterstained with eosin for 1–2 min. Finally, the sections were dehydrated through graded ethanol, cleared in xylene, and mounted with neutral resin. Images were captured using a light microscope.

### Immunohistochemistry

Paraffin-embedded tumor tissue sections were deparaffinized in xylene and rehydrated through a graded ethanol series. Endogenous peroxidase activity was blocked by incubation with 3% hydrogen peroxide for 10 min. Antigen retrieval was performed by autoclaving under high pressure. After rinsing with PBS, sections were blocked with goat serum for 1 h at room temperature and then incubated overnight at 4 °C with primary antibodies against HMOX1, TFRC, and POR. Following PBS washes, sections were incubated with the appropriate secondary antibodies at 37 °C for 1 h. Immunoreactivity was visualized using diaminobenzidine (DAB) substrate and counterstained with hematoxylin. After dehydration and mounting, the slides were examined under a light microscope (Olympus, Japan). For quantitative analysis, digital images were captured under identical microscope settings. For each tumor section, five randomly selected non-overlapping high-power fields were analyzed. ROIs were manually defined to include tumor areas while excluding necrotic regions and stromal components. Using Image-Pro Plus 6.0 software, a consistent color threshold was applied across all samples to identify DAB-positive staining. The integrated optical density (IOD) and positive staining area were measured, and the mean optical density (MOD = IOD/area) was calculated to represent protein expression levels. Quantification was performed in a blinded manner. In addition, staining intensity and distribution were independently evaluated by a board-certified pathologist to ensure consistency.

### Statistical analysis

All statistical analyses were performed using GraphPad Prism 8.0 software (GraphPad Software, CA, USA). Data are presented as mean ± standard deviation (SD) from at least three independent experiments, unless otherwise indicated. Comparisons between two groups were performed using an unpaired two-tailed Student’s t-test. Comparisons among multiple groups were analyzed using one-way analysis of variance (ANOVA) followed by Tukey’s multiple comparisons test. For experiments involving two independent variables, such as treatment and time, two-way ANOVA was used. Drug interaction was evaluated using the Chou–Talalay method, and the combination index (CI) was calculated using CompuSyn software. A CI value < 1 was considered synergistic, CI = 1 additive, and CI > 1 antagonistic. A p-value < 0.05 was considered statistically significant.

## Supplementary Information

Below is the link to the electronic supplementary material.

Supplementary Material 1

Supplementary Material 2

Supplementary Material 3

Supplementary Material 4

Supplementary Material 5

Supplementary Material 6

Supplementary Material 7

Supplementary Material 8

Supplementary Material 9

Supplementary Material 10

Supplementary Material 11

Supplementary Material 12

Supplementary Material 13

## Data Availability

The datasets generated or analysed during the current study are available from the corresponding author upon reasonable request.

## References

[1] Konstantinopoulos, P. A. & Matulonis, U. A. Clinical and translational advances in ovarian cancer therapy. *Nat. Cancer* **4**, 1239–1257 (2023). 37653142 10.1038/s43018-023-00617-9

[2] Gaillard, S. et al. Neoadjuvant Chemotherapy for Newly Diagnosed, Advanced Ovarian Cancer: ASCO Guideline Update. *J. Clin. Oncol.* **43**, 868–891 (2025). 39841949 10.1200/JCO-24-02589 PMC11934100

[3] Meng, Y. et al. Targeting CRL4 suppresses chemoresistant ovarian cancer growth by inducing mitophagy. *Signal. Transduct. Target. Ther.* **7**, 388 (2022). 36481655 10.1038/s41392-022-01253-y PMC9731993

[4] Zhang, J. et al. Oxidation of retromer complex controls mitochondrial translation. *Nature* **641**, 1048–1058 (2025). 40140582 10.1038/s41586-025-08756-y PMC13005050

[5] Berkel, C. & Cacan, E. Estrogen- and estrogen receptor (ER)-mediated cisplatin chemoresistance in cancer. *Life Sci.* **286**, 120029 (2021). 34634322 10.1016/j.lfs.2021.120029

[6] Rottenberg, S., Disler, C. & Perego, P. The rediscovery of platinum-based cancer therapy. *Nat. Rev. Cancer* **21**, 37–50 (2021). 33128031 10.1038/s41568-020-00308-y

[7] Berkel, C. & Cacan, E. In silico analysis of DYNLL1 expression in ovarian cancer chemoresistance. *Cell Biol. Int.* **44**, 1598–1605 (2020). 32208526 10.1002/cbin.11352

[8] Kucuk, B., Kibar, B. & Cacan, E. A broad analysis in clinical and in vitro models on regulator of G-protein signalling 10 regulation that is associated with ovarian cancer progression and chemoresistance. *Cell Biochem. Funct.* **39**, 413–422 (2021). 33354811 10.1002/cbf.3607

[9] de Souza, J. C. et al. Inflammasome activation contributes to cisplatin resistance in ovarian cancer. *J. Ovarian Res.* **18**, 294 (2025). 41388308 10.1186/s13048-025-01852-7 PMC12699907

[10] Li, H. et al. Platinum-resistant ovarian cancer: From mechanisms to treatment strategies. *Genes & Dis.* **13**, 101801 (2026). 10.1016/j.gendis.2025.101801 PMC12688694 41376855

[11] Chen, X., Kang, R., Kroemer, G. & Tang, D. Broadening horizons: The role of ferroptosis in cancer. *Nat. Rev. Clin. Oncol.* **18**, 280–296 (2021). 33514910 10.1038/s41571-020-00462-0

[12] Zhou, Q. et al. Ferroptosis in cancer: From molecular mechanisms to therapeutic strategies. *Signal. Transduct. Target. Ther.* **9**, 55 (2024). 38453898 10.1038/s41392-024-01769-5 PMC10920854

[13] Chen, Z. W. et al. Targeting GPX4 to induce ferroptosis overcomes chemoresistance mediated by the PAX8-AS1/GPX4 axis in intrahepatic cholangiocarcinoma. *Adv Sci. (Weinh)*10.1002/advs.202501042 (2025). 40391780 10.1002/advs.202501042 PMC12376697

[14] Lei, G., Zhuang, L. & Gan, B. The roles of ferroptosis in cancer: Tumor suppression, tumor microenvironment, and therapeutic interventions. *Cancer Cell.* **42**, 513–534 (2024). 38593779 10.1016/j.ccell.2024.03.011

[15] Deng, R. et al. Capsaicin orchestrates metastasis in gastric cancer via modulating expression of TRPV1 channels and driving gut microbiota disorder. *Cell. Commun. Signal.* **21**, 364 (2023). 38129926 10.1186/s12964-023-01265-3 PMC10734064

[16] Wang, Y. et al. Capsaicin Enhanced the Efficacy of Photodynamic Therapy Against Osteosarcoma via a Pro-Death Strategy by Inducing Ferroptosis and Alleviating Hypoxia. *Small* **20**, e2306916 (2024). 38221813 10.1002/smll.202306916

[17] Deng, X., Gui, Y., Zhao, L., Li, N. & Li, L. Arvanil induces ferroptosis of hepatocellular carcinoma by binding to MICU1. *Cancer Gene Ther.* **31**, 148–157 (2024). 37985721 10.1038/s41417-023-00690-3

[18] Chang, L. C. et al. Heme oxygenase-1 mediates BAY 11-7085 induced ferroptosis. *Cancer Lett.* **416**, 124–137 (2018). 29274359 10.1016/j.canlet.2017.12.025

[19] Richardson, D. L., Eskander, R. N. & O’Malley, D. M. Advances in ovarian cancer care and unmet treatment needs for patients with platinum resistance: A narrative review. *JAMA Oncol.* **9**, 851–859 (2023). 37079311 10.1001/jamaoncol.2023.0197

[20] Gao, W., Wang, X., Zhou, Y., Wang, X. & Yu, Y. Autophagy, ferroptosis, pyroptosis, and necroptosis in tumor immunotherapy. *Signal Transduct. Target Ther.* **7**, 196 (2022). 35725836 10.1038/s41392-022-01046-3 PMC9208265

[21] Sun, L. L., Linghu, D. L. & Hung, M. C. Ferroptosis: A promising target for cancer immunotherapy. *Am. J. Cancer Res.* **11**, 5856–5863 (2021). 35018229 PMC8727800

[22] Liu, Y. et al. Distinct roles of HMOX1 on tumor epithelium and macrophage for regulation of immune microenvironment in ovarian cancer. *Int. J. Surg.* **111**, 6725–6742 (2025). 40638251 10.1097/JS9.0000000000002829 PMC12527711

[23] Qiao, M. et al. ERM Inhibition Confers Ferroptosis Resistance through ROS-Induced NRF2 Signaling. *Adv. Sci. (Weinh)*. **13**, e13310 (2026). 41589654 10.1002/advs.202513310 PMC13042784

[24] Zuo, L. et al. The Nrf2-HMOX1 pathway as a therapeutic target for reversing cisplatin resistance in non-small cell lung cancer via inhibiting ferroptosis. *Cell Death Discov.* **11**, 287 (2025). 40544155 10.1038/s41420-025-02564-z PMC12182566

[25] Amaya-Rodriguez, C. A., Carvajal-Zamorano, K., Bustos, D., Alegria-Arcos, M. & Castillo, K. A journey from molecule to physiology and in silico tools for drug discovery targeting the transient receptor potential vanilloid type 1 (TRPV1) channel. *Front. Pharmacol.* **14**, 1251061 (2023). 38328578 10.3389/fphar.2023.1251061 PMC10847257

[26] Sun, M. Y. et al. Vanilloid agonist-mediated activation of TRPV1 channels requires coordinated movement of the S1-S4 bundle rather than a quiescent state. *Sci. Bull. (Beijing)* **67**, 1062–1076 (2022). 36546250 10.1016/j.scib.2022.02.016

[27] Sun, M. Y. et al. Mechanism of capsaicin entry into buried vanilloid sites in TRPV1. *Nat. Chem. Biol.* **21**, 1957–1969 (2025). 40702302 10.1038/s41589-025-01966-5

[28] Calhoon, D. et al. Glycosaminoglycan-driven lipoprotein uptake protects tumours from ferroptosis. *Nature* **644**, 799–808 (2025). 40500442 10.1038/s41586-025-09162-0 PMC12320555

[29] Ru, Q. et al. Iron homeostasis and ferroptosis in human diseases: Mechanisms and therapeutic prospects. *Signal Transduct. Target. Ther.* **9**, 271 (2024). 39396974 10.1038/s41392-024-01969-z PMC11486532

[30] Ye, P. et al. A positive-feedback loop suppresses TNBC tumour growth by remodeling tumour immune microenvironment and inducing ferroptosis. *Biomaterials* **315**, 122960 (2025). 39541840 10.1016/j.biomaterials.2024.122960

[31] Ma, M., Jiang, W. & Zhou, R. DAMPs and DAMP-sensing receptors in inflammation and diseases. *Immunity* **57**, 752–771 (2024). 38599169 10.1016/j.immuni.2024.03.002

