# Supplementary figures and images for: Arvanil reverses cisplatin resistance in ovarian cancer by activating HMOX1-driven ferroptosis
Source: Sci Rep. 2026 May 2;16:20285. doi: 10.1038/s41598-026-51046-4 (PMC13324716; doi:10.1038/s41598-026-51046-4)

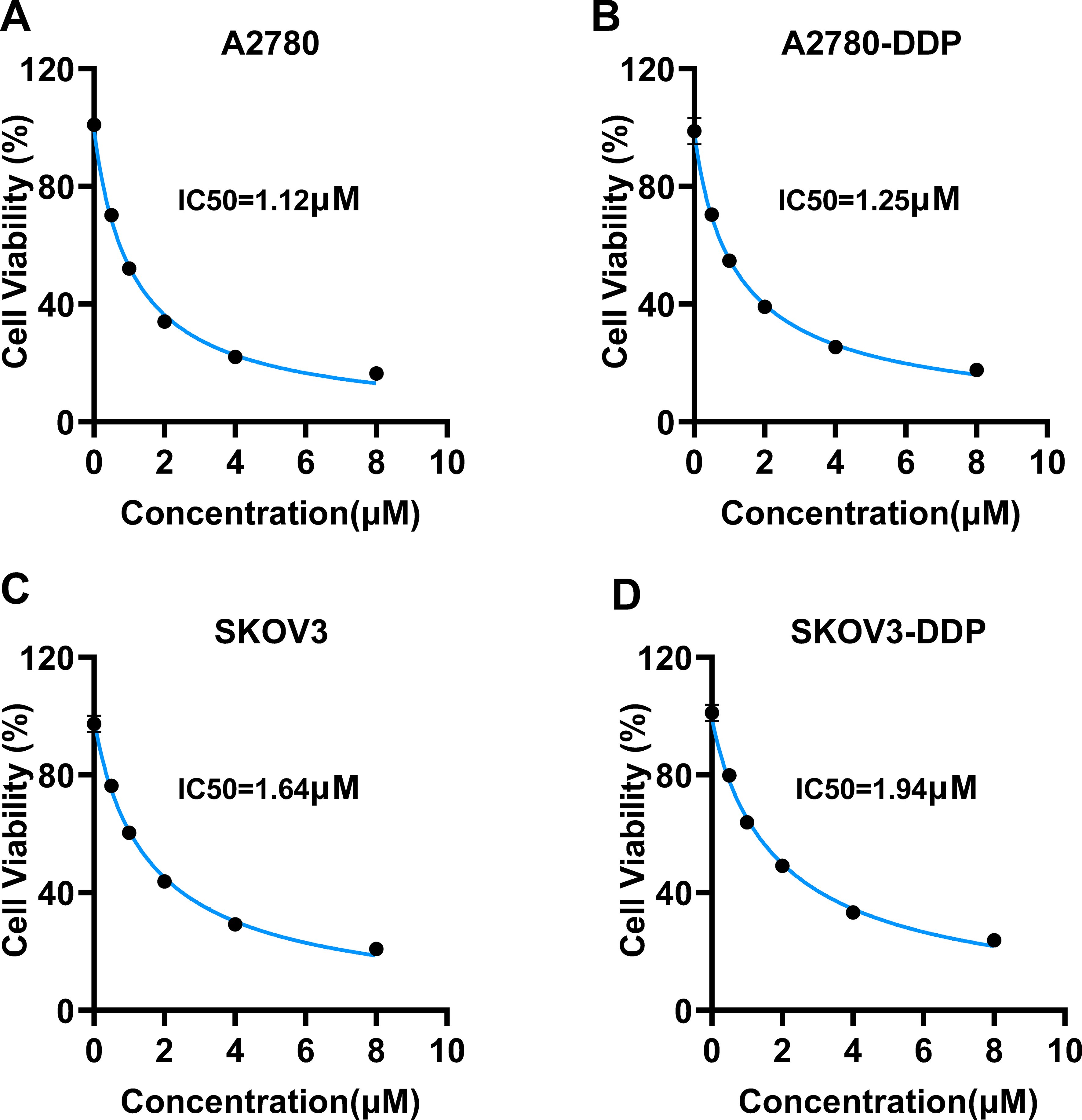

Supplement: Supplementary file 1 — Supplementary Material 1 [file 41598_2026_51046_MOESM1_ESM.tif]

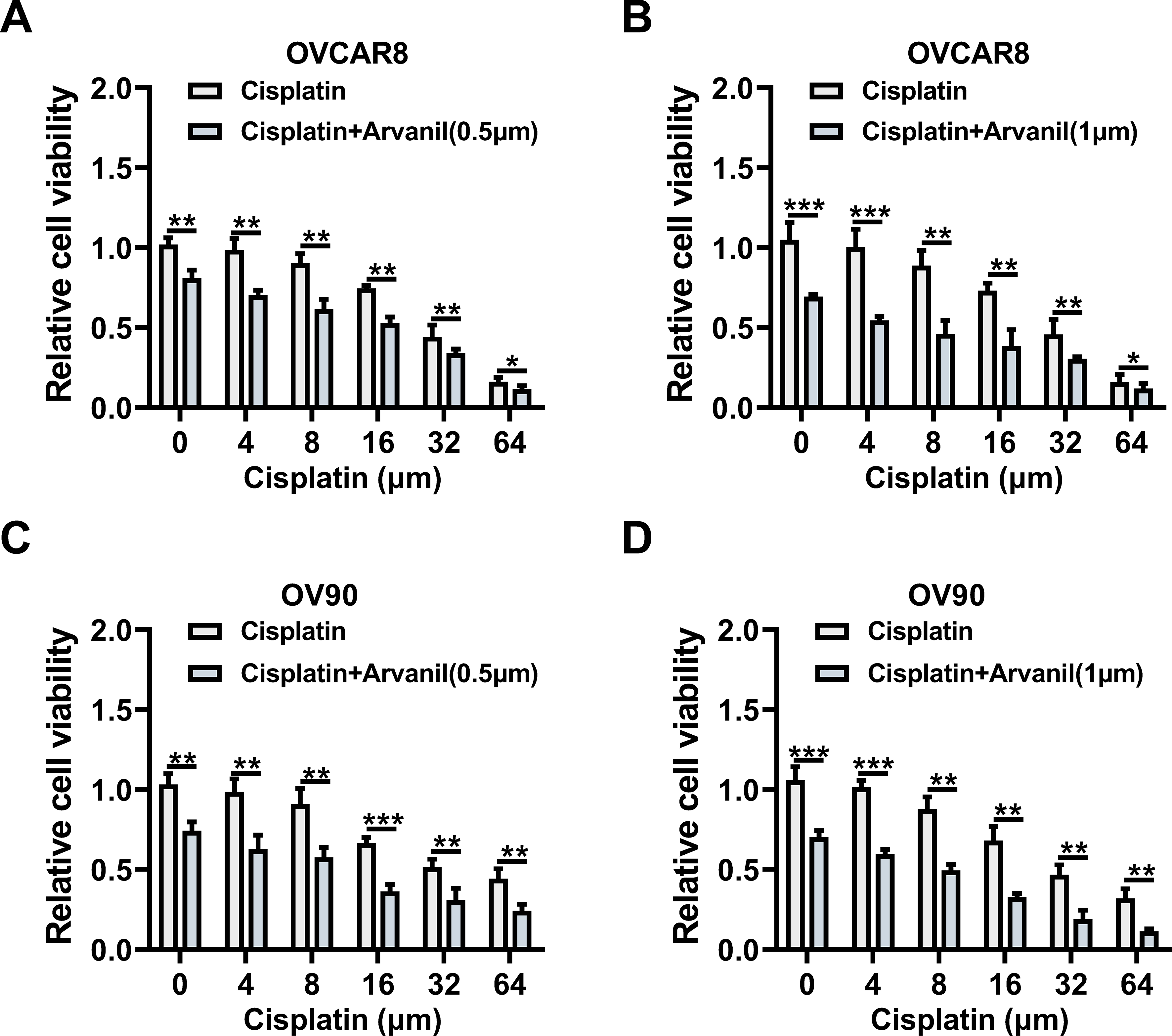

Supplement: Supplementary file 2 — Supplementary Material 2 [file 41598_2026_51046_MOESM2_ESM.tif]

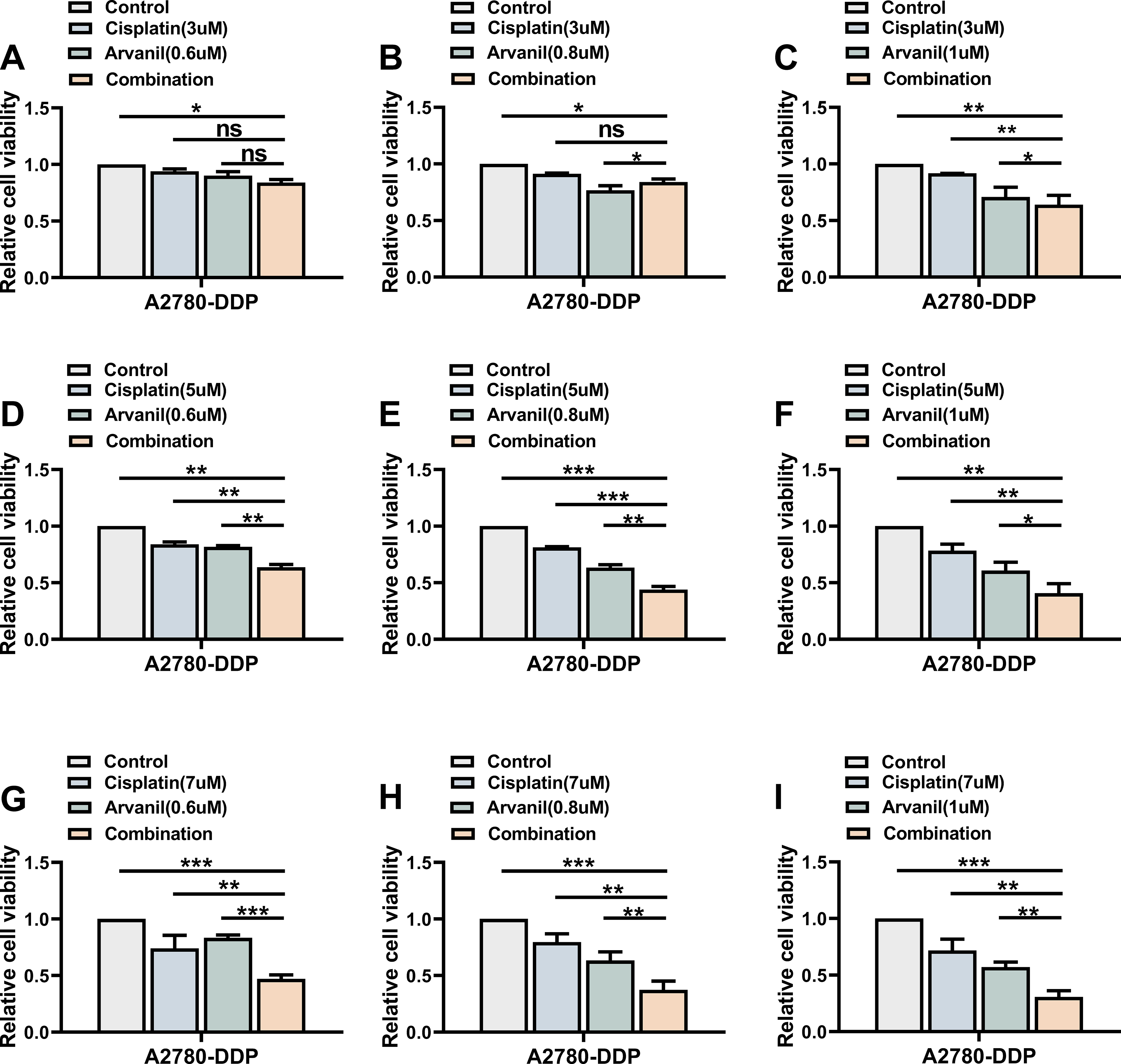

Supplement: Supplementary file 3 — Supplementary Material 3 [file 41598_2026_51046_MOESM3_ESM.tif]

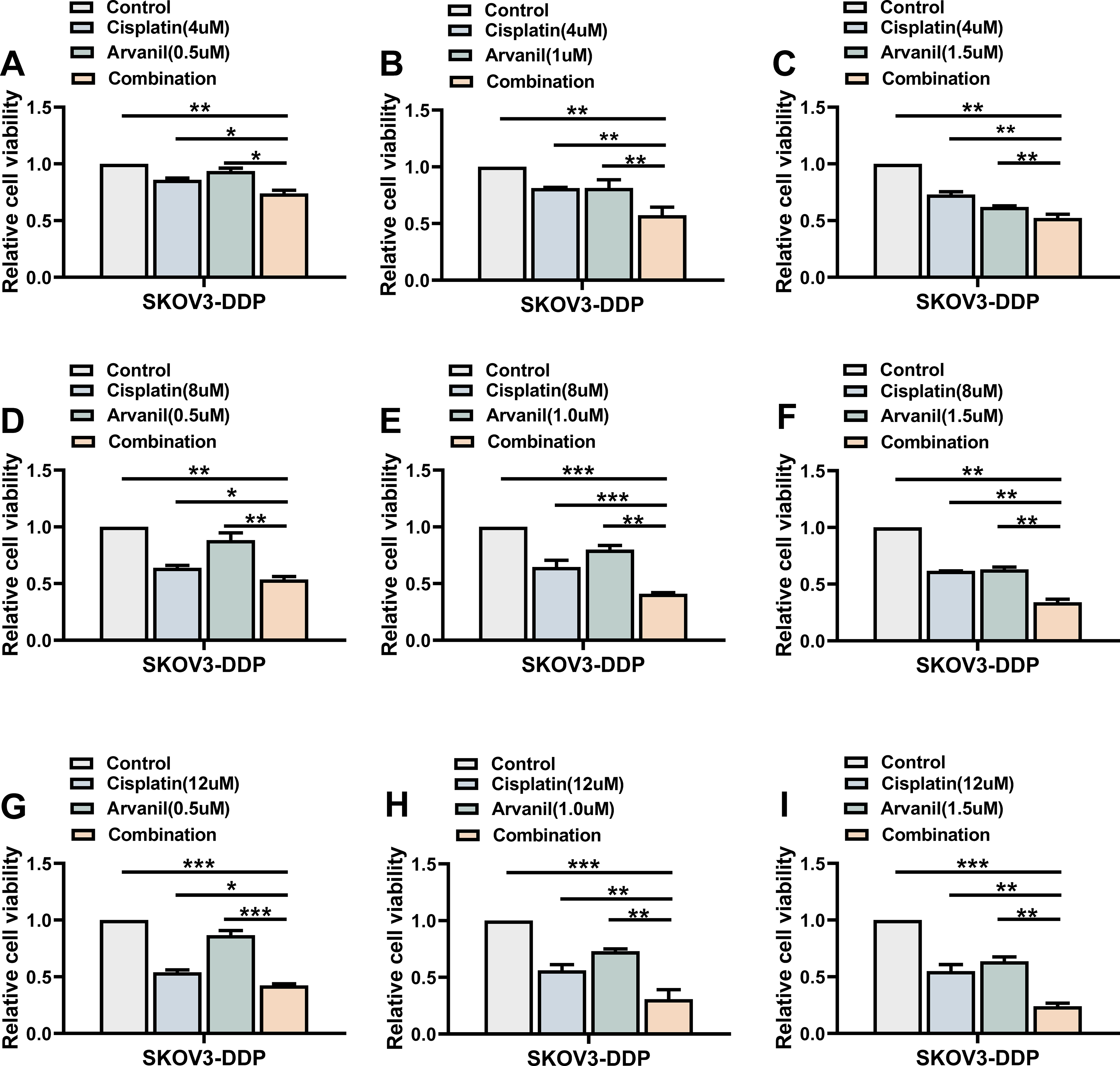

Supplement: Supplementary file 4 — Supplementary Material 4 [file 41598_2026_51046_MOESM4_ESM.tif]

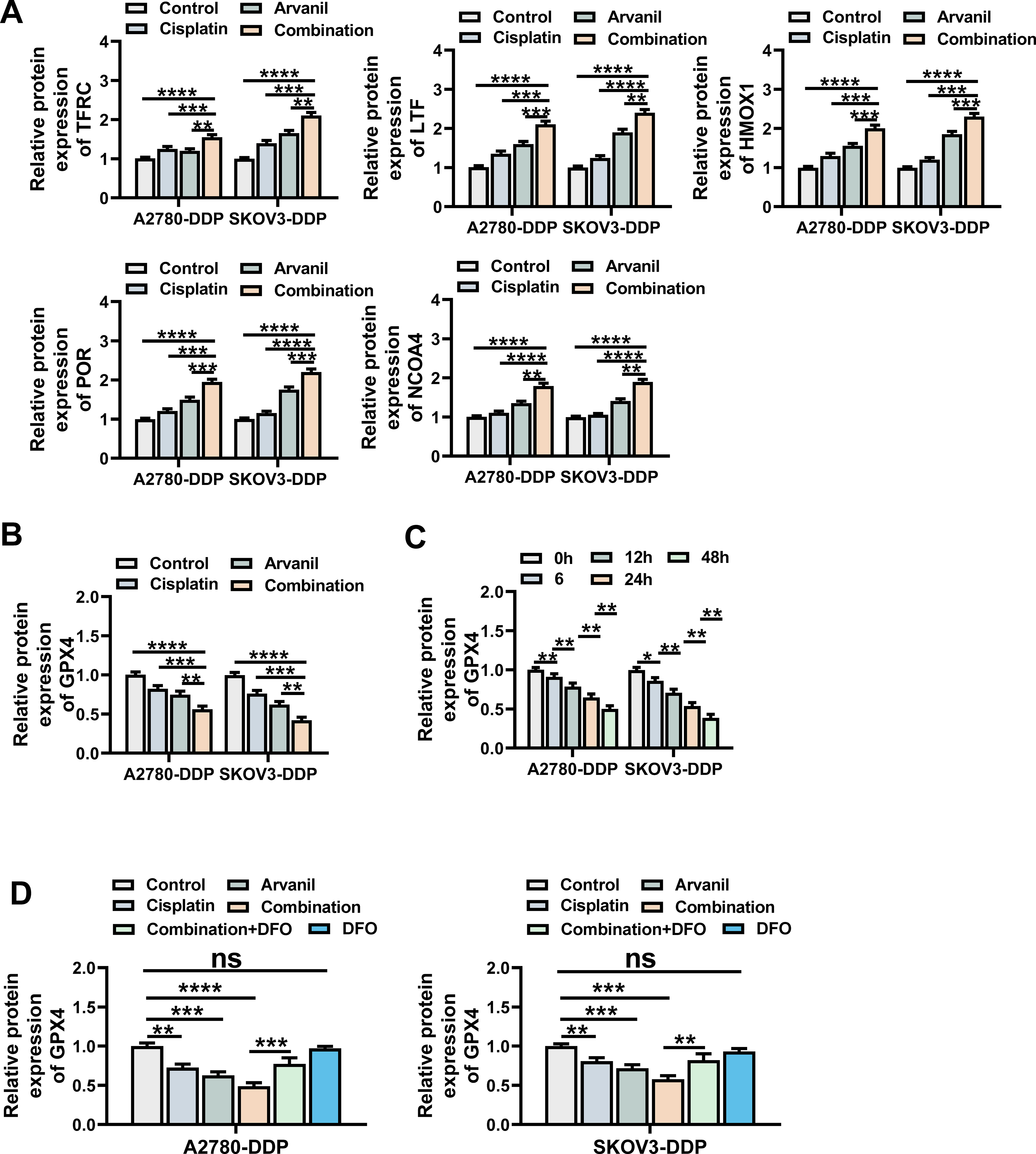

Supplement: Supplementary file 5 — Supplementary Material 5 [file 41598_2026_51046_MOESM5_ESM.tif]

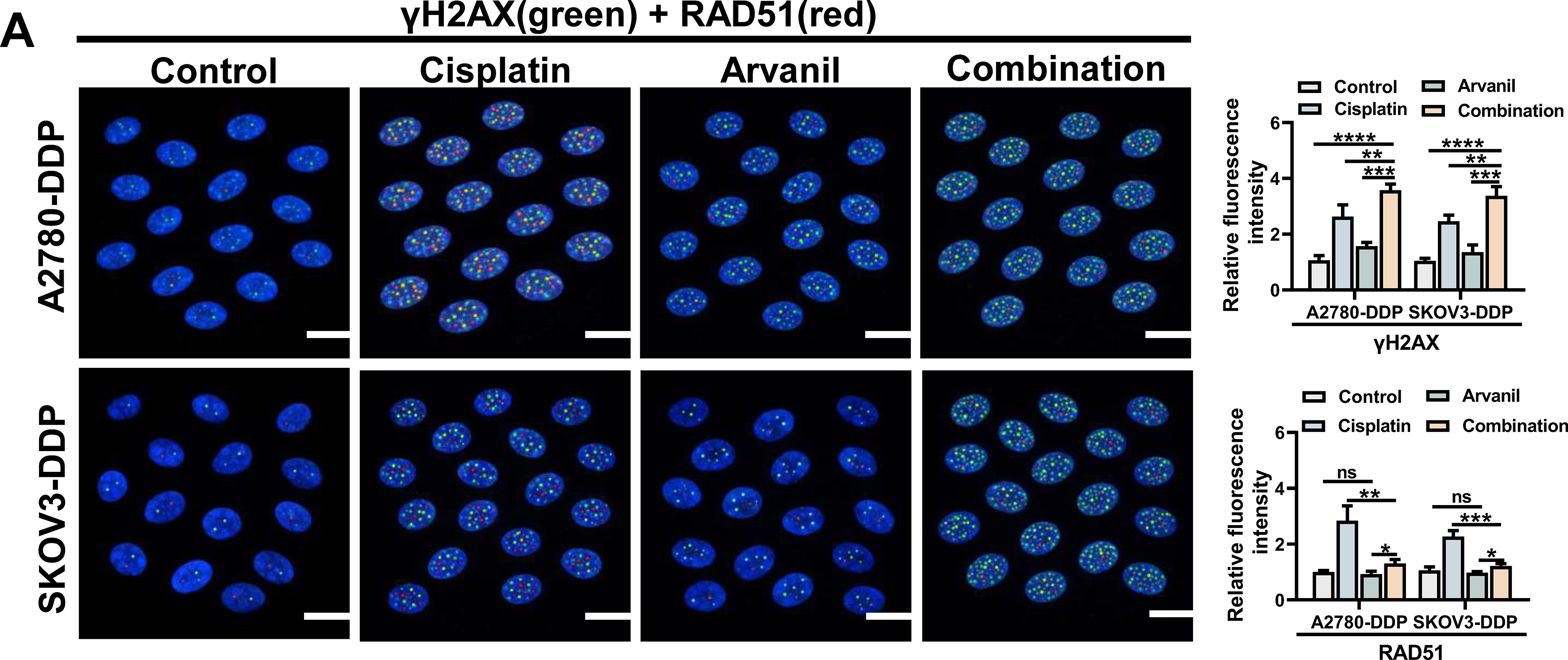

Supplement: Supplementary file 6 — Supplementary Material 6 [file 41598_2026_51046_MOESM6_ESM.tif]

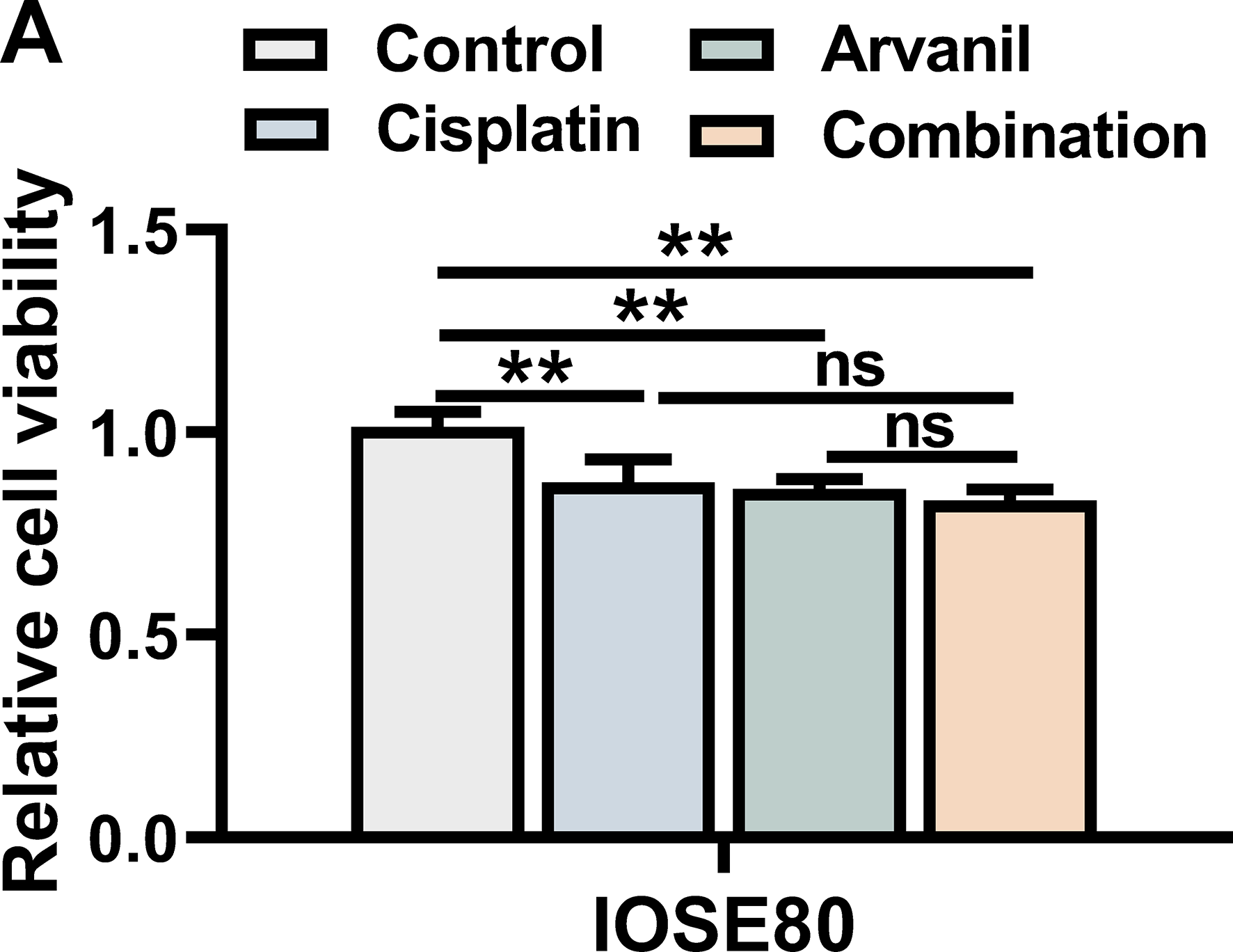

Supplement: Supplementary file 7 — Supplementary Material 7 [file 41598_2026_51046_MOESM7_ESM.tif]

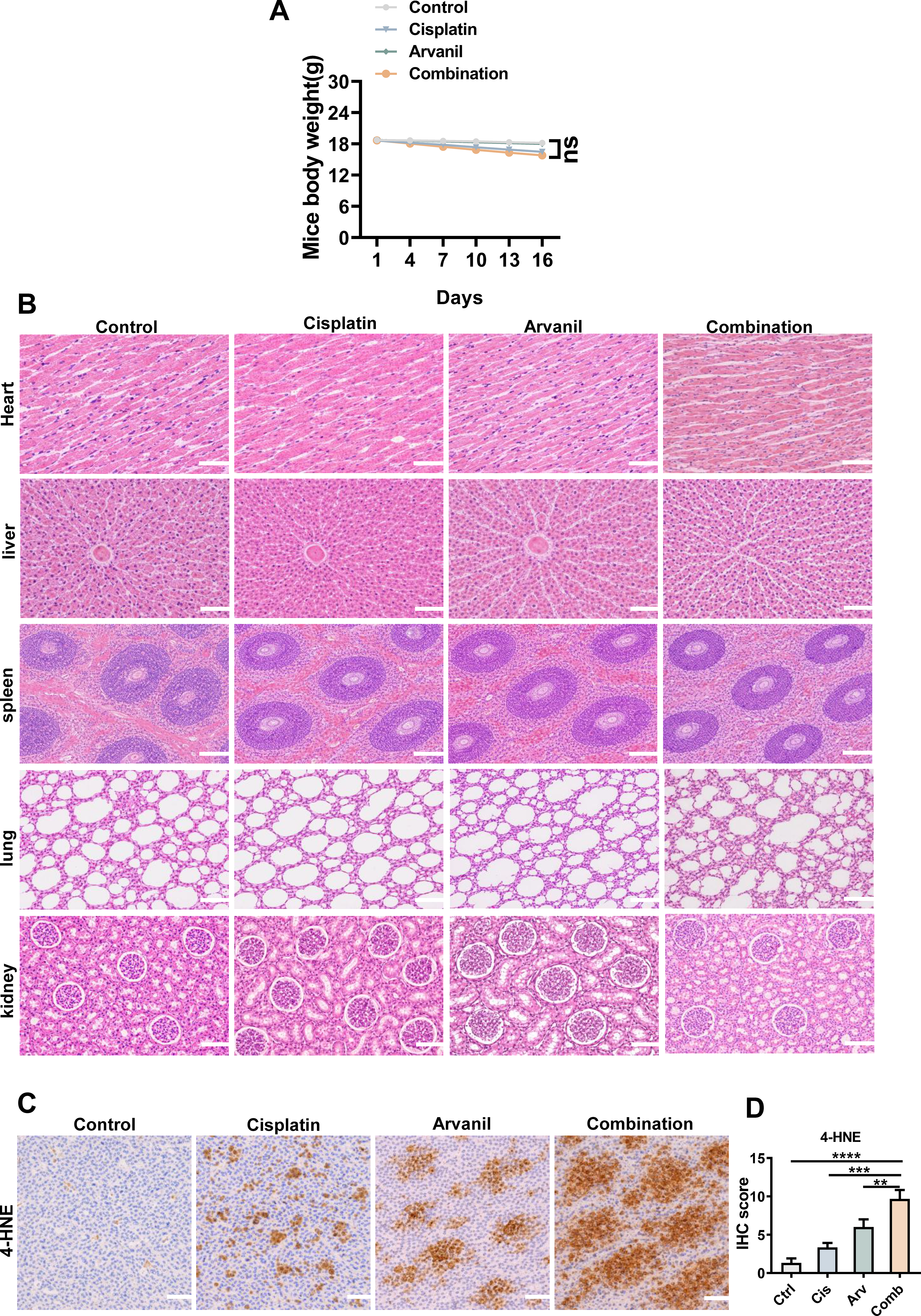

Supplement: Supplementary file 8 — Supplementary Material 8 [file 41598_2026_51046_MOESM8_ESM.tif]
