# Supplementary material for: Arvanil reverses cisplatin resistance in ovarian cancer by activating HMOX1-driven ferroptosis
Source: Sci Rep. 2026 May 2;16:20285. doi: 10.1038/s41598-026-51046-4 (PMC13324716; doi:10.1038/s41598-026-51046-4)

Original western blot images

Fig 5A **Group:** Control, Cisplatin, Arvanil, Cisplatin+ Arvanil

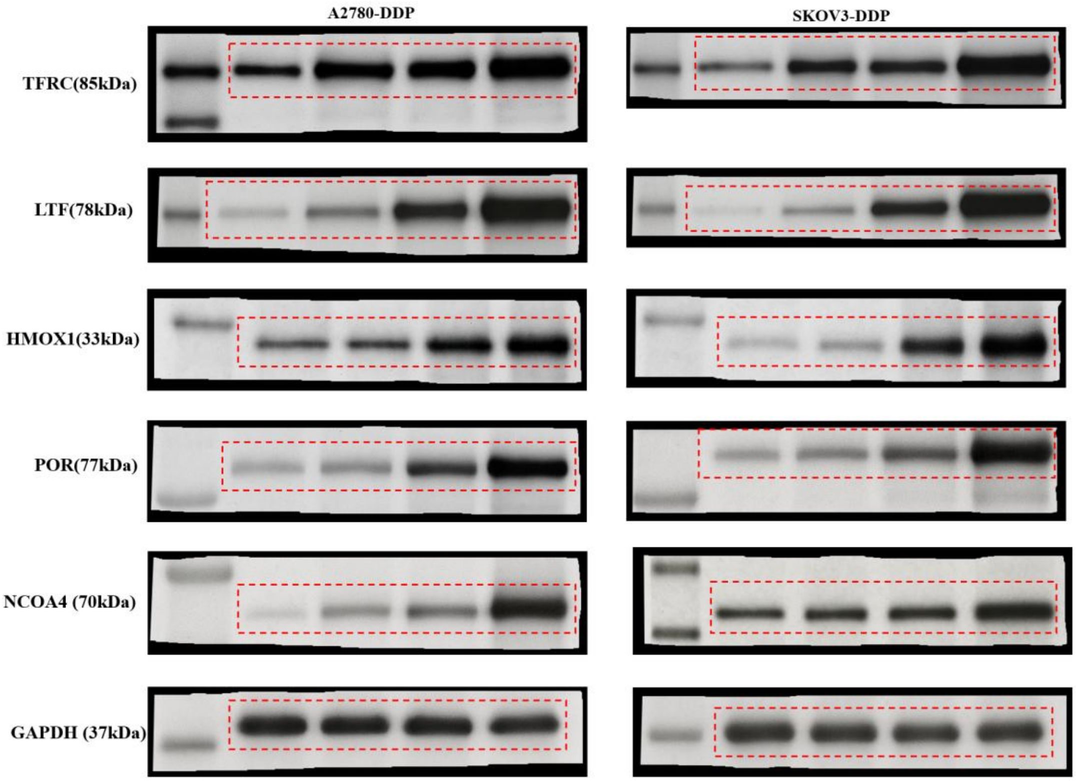

Fig 5B **Group:** Control, Cisplatin, Arvanil, Cisplatin+ Arvanil

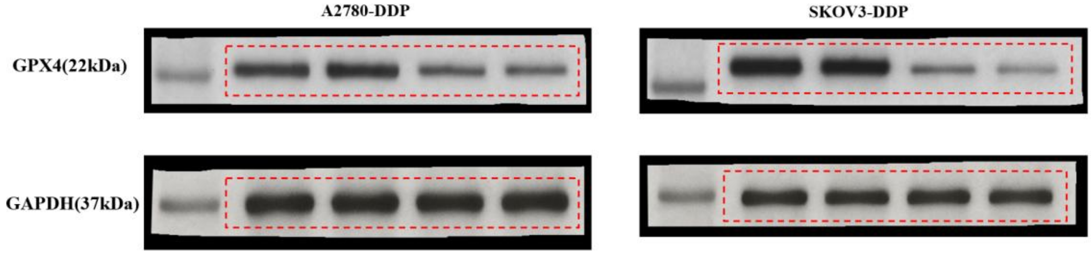

Fig 5C **Group:** Cisplatin+ Arvanil(0,6,12,24,48h)

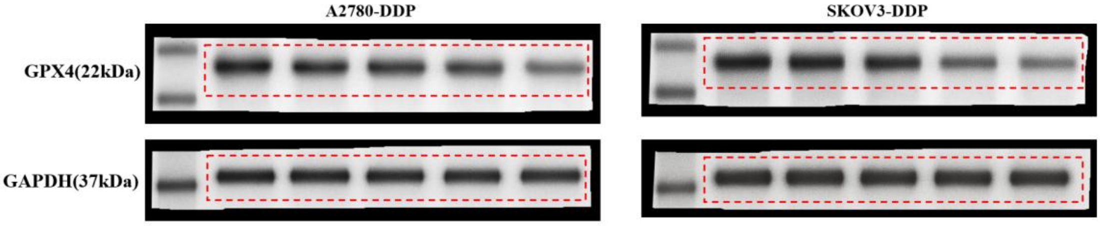

Fig 5D **Group:** Control, Cisplatin, Arvanil, Cisplatin+ Arvanil, Cisplatin+ Arvanil+DFO,DFO

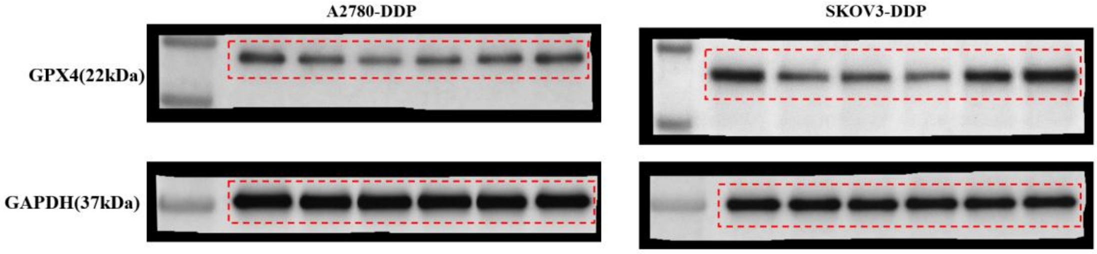

Supplement: Supplementary file 10 — Supplementary Material 10 [file 41598_2026_51046_MOESM10_ESM.pdf]
