# Supplementary material for: Arvanil reverses cisplatin resistance in ovarian cancer by activating HMOX1-driven ferroptosis
Source: Sci Rep. 2026 May 2;16:20285. doi: 10.1038/s41598-026-51046-4 (PMC13324716; doi:10.1038/s41598-026-51046-4)
Supplement: Supplementary file 12 — Supplementary Material 12 [file 41598_2026_51046_MOESM12_ESM.docx]

**Arvanil Reverses Cisplatin Resistance in Ovarian Cancer by Activating HMOX1-Driven Ferroptosis**

**Table S1. A list of qRT-PCR primers used in this study**

| **Primer** | **Sequence** |
| --- | --- |
| HMOX1-F | 5'-AACTTTCAGAAGGGCCAGGT-3' |
| HMOX1-R | 5'-GTAGACAGGGGCGAAGACTG-3' |
| TFRC-F | 5'-AAAATCCGGTGTAGGCACAG-3' |
| TFRC-R | 5'-CACCAACCGATCCAAAGTCT-3' |
| LTF-F | 5'-TGAGAATGCTGGAGACGTTG-3' |
| LTF-R | 5'-TCTGCCAGCTTCAAATCCTT-3' |
| POR-F | 5'-CCCTTCATAGGCTTCATCCA-3' |
| POR-R | 5'-CCCGGTACAGGTAGTCCTCA-3' |
| NCOA4-F | 5'-GCAAACCTGCCAGTGGTTAT-3' |
| NCOA4-R | 5'-AATTGCAGGCTCTGGAAGAA-3' |
| GPX4-F | 5'-GGACACCGTCTCTCCACAGT-3' |
| GPX4-R | 5'-GGGGCAGGTCCTTCTCTATC-3' |
| β-actin-F | 5'-TCACCAACTGGGACGACATG -3' |
| β-actin-R | 5'-GTCACCGGAGTCCATCACGAT -3' |

F: Forward primer; R: Reverse primer

**Table S2 siRNA sequence**

| **Name** | **sequence** |
| --- | --- |
| si-NC | 5'-UUUAGUAGUACAUAAUGUAGA-3' |
| si- HMOX1 -#1 | 5'- AGAAUCUUGCACUUUGUUGCU -3' |
| si- HMOX1 -#2 | 5'- AUUCACAUGGCAUAAAGCCCU -3' |
