# Supplementary material for: Arvanil reverses cisplatin resistance in ovarian cancer by activating HMOX1-driven ferroptosis
Source: Sci Rep. 2026 May 2;16:20285. doi: 10.1038/s41598-026-51046-4 (PMC13324716; doi:10.1038/s41598-026-51046-4)
Supplement: Supplementary file 13 — Supplementary Material 13 [file 41598_2026_51046_MOESM13_ESM.docx]

**Figure S1. IC₅₀ values of Arvanil in OC cell lines.** The IC₅₀ values of Arvanil were determined in parental OC cell lines (A2780 and SKOV3) and their cisplatin-resistant counterparts (A2780-DDP and SKOV3-DDP) using the CCK-8 assay after 48 h of treatment. A-D. Dose-response curves were generated, and IC₅₀ values were calculated based on cell viability relative to untreated controls.

**Figure S2. Effects of Arvanil on intrinsically cisplatin-resistant OC cell lines.** (A–D) Cell viability in intrinsically cisplatin-resistant cell lines was measured after 48 h of treatment with the indicated concentrations of cisplatin, Arvanil, or their combination. **p* < 0.05; ***p*< 0.01; ****p*< 0.001; *****p*< 0.0001; ns, *p* > 0.05.

**Figure S3. Cell viability of A2780-DDP cells treated with cisplatin and Arvanil alone or in combination for 48 hours.** Cells were exposed to varying concentrations of cisplatin and Arvanil for 48 h, and viability was assessed using the CCK-8 assay. **p* < 0.05; ***p*< 0.01; ****p*< 0.001; *****p*< 0.0001; ns, *p* > 0.05.

**Figure S4. Cell viability of SKOV3-DDP cells treated with cisplatin and Arvanil alone or in combination for 48 hours.** Cells were treated with varying concentrations of cisplatin and Arvanil for 48 h, and cell viability was evaluated using the CCK-8 assay. **p* < 0.05; ***p*< 0.01; ****p*< 0.001; *****p*< 0.0001; ns, *p* > 0.05.

**Figure S5. Effects of Arvanil and cisplatin on ferroptosis-related protein expression in cisplatin-resistant ovarian cancer cells.** (A) Relative protein expression levels of TFRC, LTF, HMOX1, POR, and NCOA4 in A2780-DDP and SKOV3-DDP cells treated with Control, Arvanil, cisplatin, or their combination. (B) Relative protein expression of GPX4 in A2780-DDP and SKOV3-DDP cells under the indicated treatments. (C) Time-dependent changes in GPX4 protein expression in A2780-DDP and SKOV3-DDP cells at 0, 6, 12, 24, and 48 h after treatment. (D) Relative protein expression of GPX4 in A2780-DDP and SKOV3-DDP cells treated with Control, Arvanil, cisplatin, combination, combination + DFO, or DFO alone. **p* < 0.05; ***p*< 0.01; ****p*< 0.001; *****p*< 0.0001; ns, *p* > 0.05.

**Figure S6. Immunofluorescence staining of γH2AX and RAD51 in cisplatin-resistant OC cells.** Cisplatin-resistant OC cells were treated with cisplatin, Arvanil, or their combination for the indicated time periods, followed by immunofluorescence staining of γH2AX and RAD51. Representative confocal images show γH2AX (green) and RAD51 (red) signals, with nuclei counterstained with DAPI (blue). All images were acquired using identical imaging parameters. **p* < 0.05; ***p*< 0.01; ****p*< 0.001; *****p*< 0.0001; ns, *p* > 0.05.

**Figure S7. Effects of Arvanil and cisplatin on cell viability in human normal ovarian epithelial cells.** Human normal ovarian epithelial IOSE80 cells were treated with cisplatin, Arvanil, or their combination at the indicated concentrations for 48 h, and cell viability was assessed using the CCK-8 assay. **p* < 0.05; ***p*< 0.01; ****p*< 0.001; *****p*< 0.0001; ns, *p* > 0.05.

**Figure S8. Evaluation of systemic toxicity and lipid peroxidation in vivo.** (A) Body weight of mice in different treatment groups was recorded during the treatment period. (B) Representative H&E staining images of major organs, including heart, liver, spleen, lung, and kidney, collected from each group. (C) IHC staining images of 4-hydroxynonenal (4-HNE) in tumor tissues from each group. (D) Quantification of 4-HNE IHC staining in tumor tissues. **p* < 0.05; ***p*< 0.01; ****p*< 0.001; *****p*< 0.0001; ns, *p* > 0.05.
